# Prevalence of HCV genotypes and subtypes in Southeast Asia: A systematic review and meta-analysis

**DOI:** 10.1371/journal.pone.0251673

**Published:** 2021-05-20

**Authors:** Ahmad Adebayo Irekeola, Nurul Adila Malek, Yusuf Wada, Nazri Mustaffa, Nur Izat Muhamad, Rafidah Hanim Shueb

**Affiliations:** 1 Department of Medical Microbiology and Parasitology, School of Medical Sciences, Universiti Sains Malaysia, Health Campus, Kubang Kerian, Kelantan, Malaysia; 2 Microbiology Unit, Department of Biological Sciences, College of Natural and Applied Sciences, Summit University Offa, Offa Kwara State, Nigeria; 3 Department of Pathology, Hospital Sultanah Nur Zahirah, Kuala Terengganu, Terengganu, Malaysia; 4 Department of Zoology, Faculty of Life Sciences, Ahmadu Bello University, Zaria, Nigeria; 5 Department of Medicine, School of Medical Sciences, Universiti Sains Malaysia, Health Campus, Kubang Kerian, Kelantan, Malaysia; 6 Institute for Research in Molecular Medicine (INFORMM), Universiti Sains Malaysia, Kubang Kerian, Kelantan, Malaysia; Centre de Recherche en Cancerologie de Lyon, FRANCE

## Abstract

Known for its high genetic diversity and variation in genotypic presence in different regions of the world, hepatitis C virus (HCV) is estimated to infect about 71 million people globally. Selection of an appropriate therapeutic regimen largely depends on the identification of the genotype responsible for the infection. This systematic review and meta-analysis was conducted to provide a comprehensive view of HCV genotype and subtype distribution in Southeast Asia (SEA). The review was conducted according to the Preferred Reporting Items for Systematic Reviews and Meta-analysis (PRISMA). We searched five databases without year and language restrictions. Data from 90 eligible studies involving 15,089 genotypes and 9,646 subtypes representing 10 SEA countries were analyzed. The pooled estimates showed that genotype 1 (46.8%) [95% CI, 43.2–50.4; *I*^*2*^ = 92.77%; *p* < 0.001] was the most dominant HCV genotype in the region, followed by genotype 3 (23.1%) [95% CI, 19.4–27.2; *I*^*2*^ = 93.03%; *p* < 0.001], genotype 6 (16.5%) [95% CI, 13.8–19.6], genotype 2 (4.6%) [95% CI, 3.5–5.9], genotype 4 (1.1%) [95% CI, 0.7–1.5] and genotype 5 (0.8%) [95% CI, 0.4–1.3]. Philippines had the highest prevalence of genotypes 1 and 2. Genotype 6 became more prevalent after year 2000. Over 40 different subtypes were identified, with subtypes 1b (26.3%), 1a (21.3%), and 3a (14.3%) being the most prevalent of all the reported subtypes. Although on a global scale, genotype 6 is considered highly prevalent in SEA, evidence from this study reveals that it is the third most prevalent genotype within the region.

## Introduction

Hepatitis C virus (HCV) infection constitutes a major health challenge globally. HCV is a principal cause of hepatocellular carcinoma (HCC), liver cirrhosis and liver failure, and it is thought to infect about 71 million people worldwide [1]. The virus, which belongs to the *Flaviviridae* family, consists of a positive-sense single-stranded RNA genome that spans approximately 9.6 kb [2, 3]. Flanked by 5’ and 3’ untranslated regions (UTRs), its long single open reading frame codes for structural (core [C] and envelope [E1 and E2]) and non-structural (P7, NS2, NS3, NS4A, NS4B, NS5A and NS5B) proteins [2, 3]. HCV exhibits a profound genetic diversity–an occurrence partly attributed to the absence of proofreading nuclease activity of its ribonucleic acid (RNA)-dependent RNA polymerase [4, 5]. Similar to human immunodeficiency virus (HIV), multiple quasispecies of HCV emerge quite frequently in vivo, posing significant threat to drug efficacy and vaccine development. Mutations that occur in the least conserved regions (i.e., hypervariable regions of the NS5A and envelope genes) of the HCV viral genome are thought to play a crucial role in immune evasion and the establishment of chronic infection [5].

Sequence and phylogenetic analysis of HCV genome has revealed at least six major genotypes (designated as genotype 1 to 6) and numerous subtypes (connoted with lowercase alphabets; e.g. 1a, 1b, 2a, etc.) [6]. Although HCV exhibits an extraordinary sequence diversity, all genotypes possess identical complement of co-linear genes of similar size within the open reading frame (ORF), and the known variants contain considerably similar sequence across the genome [7]. This makes it possible to classify them based on partial sequences from specific regions (e.g. core/E1 or NS5B) within the genome [7]. The distribution of HCV genotypes is largely dependent on geographical locations. Although genotypes 1, 2 and 3 seem to be distributed globally, ‘endemic’ strains of genotype 1 and 2 are mainly in West Africa while genotype 3 is dominant in South Asia [4]. Genotypes 4, 5, and 6 on the other hand, are predominant in the Middle East and Central Africa, Southern Africa, and Southeast Asia, respectively [4].

Distinct genotypes and subtypes of HCV vary in their degrees of resistance to some of the currently available antiviral drugs, making treatment decision genotype oriented [6, 8]. Determination of infecting genotype is thus fundamental to HCV therapy as it helps ascertain which antiviral drug to administer, its dosage, as well as treatment duration [9]. While there are several HCV genotyping methods, full genomic analysis or sequencing and phylogenetic analysis of informative and conserved regions (e.g. core/E1 or NS5B) of the genome remains the gold standard [5, 6, 10].

In the past, attempts have been made to provide global and regional distribution of HCV genotypes [4, 11–13]. However, there has been no detailed report on the actual distribution of the virus’ genotypes in Southeast Asia (SEA)–a multiethnic and socioculturally diverse Asian region spanning Brunei, Cambodia, East Timor, Indonesia, Laos, Malaysia, Myanmar, Philippines, Singapore, Thailand and Vietnam. This is further compounded by the fact that newer variants of the virus are continually being identified and characterized. In this article, we conducted a Preferred Reporting Items for Systematic Reviews and Meta-analysis (PRISMA) compliant review of published articles reporting HCV genotypes in SEA since the virus was first identified in 1989 to provide an in-depth and up-to-date information on the distribution of HCV genotypes in the region.

## Methods

### Search strategy and selection criteria

We accessed five electronic databases (PubMed, Scopus, ScienceDirect, Google Scholar, and Web of Science) for studies reporting HCV genotypes in eleven SEA countries (Brunei, Cambodia, East Timor, Indonesia, Laos, Malaysia, Myanmar, Philippines, Singapore, Thailand, Vietnam). The databases were searched using a combination of terms (e.g., “hepatitis c”, “genotype”) related to the distribution of HCV genotypes in SEA without year or language restrictions. Details of the search strategy used is provided in **S1 File**. A final search was made on April 30, 2020.

Studies that conducted HCV genotyping and whose samples or analyzed data were collected in at least one of the SEA countries were included. We excluded studies (1) that are reviews, (2) with unknown sample origins (3) with only serological findings, (4) whose full text could not be obtained, or (5) contained duplicated data.

### Quality assessment

The methodological quality of all the included studies was assessed using the Joanna Briggs Institute (JBI) critical appraisal checklist for prevalence data [14] (**S2 File**). A score of ‘1’ for ‘yes’ and ‘0’ for other parameters were assigned to attain a total quality score that ranged from 0 to 9. Studies with overall score of 7–9 were considered to be of sufficient quality. Two authors (A.A.I and Y.W.) independently assessed the studies. Studies were included if there was a consensus between the two reviewers. The quality assessment of the 90 included studies is provided in **S1 Table**.

### Data extraction and analysis

From each of the included studies, three authors (A.A.I., N.A.M., and Y.W.) independently extracted information regarding country, year of publication, sample size, year of sample collection as well as reported HCV genotypes and subtypes. Data was also extracted for HCV genotypes reported without details of the subtypes. Disagreements were resolved by discussion and adjudication including a fourth author (R.H.S.). We converted HCV genotypes and variants published prior to 1994 to the consensus nomenclature proposed by Simmonds et al. [15] since a standardized genotype classification system was not available in the earlier years of HCV research. Where more than one article reported data from the same sample, record, or patient cohort, only one was counted and selected. Similarly, studies that genotyped and analyzed samples from more than one SEA country were categorized as “multi-country” rather than the individual countries included, and the data were extracted and analyzed in that form to avoid confusion. Data on ‘inconclusive’ or ‘untyped/undetermined’ genotypes and/or subtypes were neither extracted nor analyzed. Cases of more than one genotype and/or subtype from a single patient were tagged ‘mixed.’ Although identified, these mixed genotype data were excluded from the genotype and subtype analysis. In the case of immigrant workers, irrespective of their original countries, their data were pooled to reflect the countries they were working in when their samples were collected.

Data analysis was conducted using OpenMeta Analyst and meta (version 4.15–1) and metafor (version 2.4–0) packages of R (version 4.0.3) and RStudio (version 1.3.1093) [16]. The pooled prevalence of HCV genotypes was calculated, and subgroup analysis was done according to country and period of data collection. Random-effect model using the DerSimonian-Laird method of meta-analysis was employed to determine the pooled estimates of the reported HCV genotype and subtype proportions. Data were transformed using the logit transformation. A forest plot was subsequently generated to visually summarize details of the individual studies alongside the estimated common effect and degree of heterogeneity. Publication bias was examined using funnel plots (visual aid for detecting bias) and Egger’s regression test. The heterogeneities (i.e., variation in study outcomes between studies) of study-level estimates were evaluated by Cochran’s Q test and quantified using *I*^*2*^ statistics. *I*^*2*^ values of 25%, 50%, and 75% were considered low, moderate, and high heterogeneity, respectively [17]. Subgroup meta-analysis was used to analyze sources of heterogeneity. Sensitivity test was conducted using the leave-one-out analysis. *P* value of < 0.001 was considered to be statistically significant for all tests. A protocol was not lodged for this study.

## Results

### Search results and eligible studies

**Fig 1:**
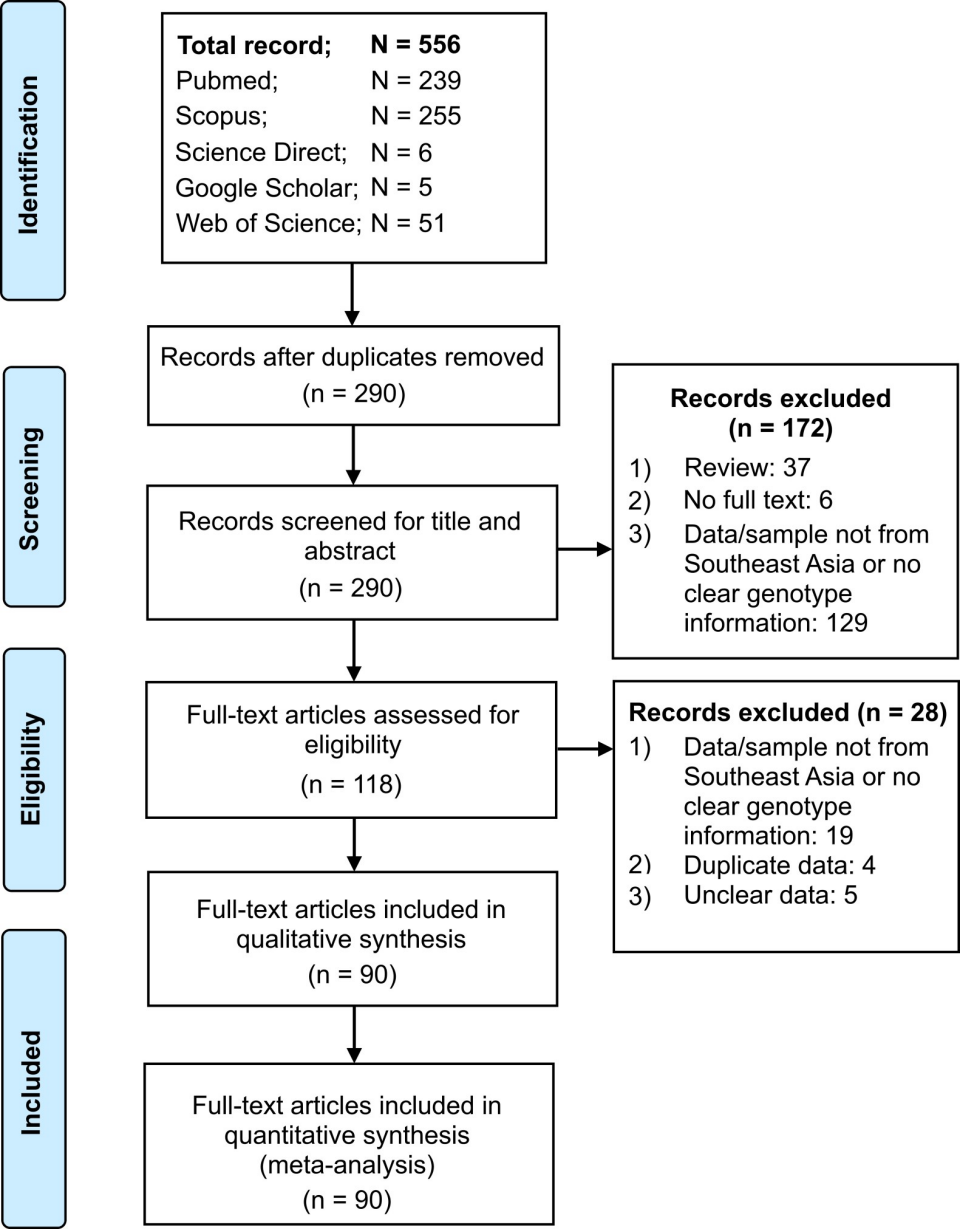
Summary of article identification and selection process.

The article selection process for this study is shown in **Fig 1**. In brief, our search of five databases returned 556 records. After duplicate removal and exclusion of studies that did not meet the inclusion criteria, a total of 90 studies were eligible and thus included in quantitative synthesis (**Fig 1**).

### Characteristics of the eligible studies

All the eligible studies were of high methodological quality (**S1 Table**). Majority of the 90 studies included in this meta-analysis were reported from Thailand (n = 27). A total of 15,089 HCV genotypes were reported across the 90 studies and ranged from 6 (in Indonesia) to 3,020 (in Cambodia) (**Table 1**). Six HCV genotypes (genotype 1 to 6) were basically reported. The most targeted regions for genotyping analysis were the 5’UTR, core and NS5B regions, meanwhile, sequencing and phylogeny were the major genotyping methods utilized. While HCV genotype information was available for all included studies, only 69 studies provided data on HCV subtypes. No study met our search criteria for East Timor. As such, genotype data analyzed included studies from ten SEA countries. The reported genotypes and subtypes varied across countries.

**Table 1:** Characteristics of the selected studies reporting HCV genotypes in Southeast Asia.

No.	Author	Country	Target region	Genotyping method	Total GT (n)	GT1 (n)	GT2 (n)	GT3 (n)	GT4 (n)	GT5 (n)	GT6 (n)
1	Chong et al., 2008 [18]	Brunei	–	sequencing	7	3	1	3	0	0	0
2	Budkowska et al., 2011 [19]	Cambodia	NS5B	phylogeny	58	29	1	0	0	0	28
3	De Weggheleire et al., 2017 [20]	Cambodia	–	LiPA	87	46	4	1	0	0	36
4	Lerolle et al., 2012 [21] *	Cambodia	NS5B	sequencing	28	19	2	0	7	0	0
5	Nouhin et al., 2019 [22]	Cambodia	NS5B	phylogeny	3020	1444	134	0	0	0	1442
6	Yamada et al., 2015 [23]	Cambodia	5’NCR	–	9	3	0	0	0	0	6
7	Anggorowati et al., 2012 [24]	Indonesia	NS5B	phylogeny	44	28	0	12	3	0	1
8	Hadikusumo et al., 2016 [25]	Indonesia	NS5B	phylogeny	6	2	0	4	0	0	0
9	Hadiwandowo et al., 1994 [26] *	Indonesia	core	PCR	81	39	24	17	0	1	0
10	Handajani et al., 2019 [27]	Indonesia	5’UTR, NS5B	phylogeny	16	9	4	3	0	0	0
11	Inoue et al., 2000 [28] ^#^	Indonesia	5’UTR	PCR, phylogeny	57	38	12	7	0	0	0
12	Juniastuti et al., 2014 [29]	Indonesia	5’UTR, NS5B	sequencing	99	57	2	39	1	0	0
13	Kurniawan et al., 2018 [30] *	Indonesia	–	–	274	199	30	32	12	0	1
14	Lesmana et al., 1996 [31]	Indonesia	5’NCR, core	PCR	104	70	33	1	0	0	0
15	Prasetyo et al., 2013 [32]	Indonesia	E1/E2, NS5B	phylogeny	29	19	0	8	2	0	0
16	Prasetyo et al., 2017 [33]	Indonesia	E1/E2	phylogeny	12	9	0	2	1	0	0
17	Prasetyo et al., 2018 [34]	Indonesia	E1/E2	phylogeny	18	14	0	3	1	0	0
18	Rinonce et al., 2013 [35]	Indonesia	NS5B	phylogeny	100	98	0	2	0	0	0
19	Sheng et al., 1994 [36] ^#^	Indonesia	–	PCR	66	51	13	0	2	0	0
20	Soetjipto et al., 1996 [37]	Indonesia	5’UTR, NS5B	sequencing	80	60	18	1	1	0	0
21	Tokita et al., 1996 [38]	Indonesia	core, NS5B	PCR, sequencing, phylogeny	126	70	42	1	0	0	13
22	Utama et al., 2008 [39]	Indonesia	5’UTR, NS5B	phylogeny	104	64	21	18	1	0	0
23	Utama et al., 2010 [40]	Indonesia	5’UTR, core, NS5B	phylogeny	150	109	24	17	0	0	0
24	Hübschen et al., 2011 [41] ^#^	Laos	core/E1, NS5B	phylogeny	45	2	0	0	0	0	43
25	Hairul et al., 2012 [42] *^,^^#^	Malaysia	5’UTR, NS5B	phylogeny	28	7	0	19	1	0	1
26	Ho et al., 2015 [43] *	Malaysia	–	linear array GT strip	396	142	7	245	0	0	2
27	Mohamed et al., 2013 [44]	Malaysia	NS5B	phylogeny	37	10	0	27	0	0	0
28	Ng et al., 2015 [45]	Malaysia	5’UTR, NS5B	phylogeny	126	52	0	72	0	0	2
29	Tan et al., 2015 [46] *	Malaysia	–	LiPA	45	12	0	33	0	0	0
30	Zheng et al., 1996 [47] ^#^	Malaysia	5’NCR, core	PCR, sequencing	7	4	0	3	0	0	0
31	Bwa et al., 2019 [48] *	Myanmar	–	LiPA	158	24	0	80	0	0	54
32	Lwin et al., 2007 [49]	Myanmar	core, NS5B	phylogeny	145	16	1	57	0	0	71
33	Naing et al., 2015 [50] *	Myanmar	NS	PCR	350	102	4	178	0	0	66
34	Nakai et al., 2001 [51]	Myanmar	5’UTR	PCR	22	4	0	18	0	0	0
35	Shinji et al., 2004 [52]	Myanmar	NS5B	phylogeny	110	35	0	52	0	0	23
36	Ye et al., 2019 [53]	Myanmar	core/E2, NS5B	phylogeny	21	3	0	9	0	0	9
37	Agdamag et al., 2005 [54]	Philippines	NS5B	phylogeny	23	15	8	0	0	0	0
38	Katayama et al., 1996 [55]	Philippines	5’UTR, NS5B	sequencing	30	27	3	0	0	0	0
39	Durier et al., 2017 [56] ^#^	Multi-country	5’UTR, NS5B	PCR	373	223	2	97	8	0	43
40	Greene et al., 1995 [57]	Multi-country	E1, E2/NS1, NS4, NS5	sequencing	57	42	2	13	0	0	0
41	Yusrina et al., 2018 [58] ^#^	Multi-country	5’UTR, core, NS5B	LiPA, PCR, phylogeny	95	28	5	41	0	0	21
42	Choy et al., 2019 [59] *	Singapore	–	LiPA	116	77	0	39	0	0	0
43	Lee et al., 1994 [60] *	Singapore	5’NCR	sequencing	40	35	2	3	0	0	0
44	Soh et al., 2019 [61]	Singapore	–	LiPA	63	24	1	27	4	0	7
45	Akkarathamrongsin et al., 2013 [62]	Thailand	core, NS5B	phylogeny	354	124	2	157	0	0	71
46	Akkarathamrongsin et al., 2011 [63]	Thailand	core	phylogeny	39	8	0	14	0	0	17
47	Avihingsanon et al., 2014 [64] *^,^^#^	Thailand	5’UTR, core, NS5B	sequencing	370	128	2	176	0	1	63
48	Barusrux et al., 2012 [65]	Thailand	5’UTR, core	sequencing	7	4	0	3	0	0	0
49	Barusrux et al., 2014 [66]	Thailand	5’UTR, core	phylogeny	101	16	0	76	0	0	9
50	Boonyarad et al., 2003 [67]	Thailand	core	LiPA, RFLP, phylogeny	7	2	0	5	0	0	0
51	Chuenjitkulthaworn et al., 2019 [68]	Thailand	–	–	20	10	0	9	0	0	1
52	Hansurabhanon et al., 2002 [69] ^#^	Thailand	–	RFLP	282	55	0	217	0	0	10
53	Jutavijittum et al., 2009 [70]	Thailand	core/E1	sequencing	124	35	0	50	0	0	39
54	Kanistanon et al., 1997 [71]	Thailand	5’UTR, core	RHA	216	76	0	99	0	0	41
55	Kumthip et al., 2014 [72]	Thailand	core	LiPA, sequencing	158	49	0	86	0	0	23
56	Luengrojanakul et al., 1994 [73] *^,^^#^	Thailand	core	PCR	83	14	21	3	0	43	2
57	Martin et al., 2019 [74]	Thailand	HVR1	phylogeny	218	94	0	93	0	0	31
58	Nakahira et al., 1995 [75] ^#^	Thailand	5’NCR	PCR	42	42	0	0	0	0	0
59	Netski et al., 2004 [76]	Thailand	core/E1	phylogeny	161	52	0	66	0	0	43
60	Sirinawasatien et al., 2019 [77] *	Thailand	core	sequencing	216	62	0	110	0	0	44
61	Sirinawasatien et al., 2020 [78] *	Thailand	core	sequencing	185	74	0	70	0	0	41
62	Sistayanarain et al., 2011 [79] ^#^	Thailand	core	PCR	100	28	0	40	0	0	32
63	Smolders et al., 2018 [80] *^,^^#^	Thailand	–	–	98	38	0	46	0	0	14
64	Songsivilai et al., 1996 [81]	Thailand	5’NCR	RHA	8	3	0	5	0	0	0
65	Sugiyama et al., 1995 [82]*	Thailand	5’UTR, core, NS5	PCR sequencing	13	7	2	4	0	0	0
66	Sunanchaikarn et al., 2007 [83]	Thailand	5’UTR, core	sequencing	45	15	2	24	0	0	4
67	Theamboonlers et al., 2000 [84]	Thailand	5’NCR, core	RFLP	124	61	0	54	0	0	9
68	Tokita et al., 1995 [85] ^#^	Thailand	core	PCR, sequencing, phylogeny	79	33	3	43	0	0	0
69	Wasitthankasem et al., 2015 [86]	Thailand	core, NS5B	phylogeny	588	191	3	271	0	0	123
70	Wasitthankasem et al., 2016 [87]	Thailand	core, NS5B	phylogeny	23	4	0	11	0	0	8
71	Wasitthankasem et al., 2017 [88]	Thailand	core	phylogeny	234	74	0	73	0	0	87
72	Do et al., 2015 [89]	Vietnam	–	–	9	1	1	1	0	0	6
73	Dunford et al., 2012 [90]	Vietnam	core/E1, NS5B	phylogeny	282	169	1	5	0	0	107
74	Duong et al., 2016 [91]	Vietnam	–	genotyping kit	14	10	0	0	0	0	4
75	Duong et al., 2019 [92]	Vietnam	–	genotyping kit	18	13	0	0	0	0	5
76	Kakumu et al., 1998 [93] ^#^	Vietnam	core	PCR	42	22	8	2	0	0	0
77	Le Ngoc et al., 2019 [94]	Vietnam	5’UTR, core, NS5B	phylogeny	322	182	24	7	0	0	109
78	Li et al., 2014 [95]	Vietnam	core/E1, NS5B	phylogeny	236	77	34	0	0	0	125
79	Lioznov et al., 2016 [96] *	Vietnam	–	–	1604	806	308	0	0	0	490
80	Minh 2015 [97] *	Vietnam	NS5B	phylogeny	390	111	61	2	0	0	216
81	Nadol et al., 2016 [98] *	Vietnam	5’UTR	phylogeny	41	30	1	0	0	0	10
82	Nguyen et al., 2016 [99]	Vietnam	core, NS5B	sequencing	93	64	0	5	0	0	24
83	Nguyen et al., 2018 [100] *	Vietnam	–	–	82	57	0	3	0	0	22
84	Noppornpanth et al., 2006 [101] *^,^^#^	Vietnam	core, NS5B	phylogeny	58	22	6	0	0	0	30
85	Pham et al., 2009 [102]	Vietnam	core, NS5B	phylogeny	70	33	0	4	0	0	33
86	Pham et al., 2011 [103]	Vietnam	NS5B	sequencing	842	256	128	0	0	0	458
87	Song et al., 1994 [104] ^#^	Vietnam	core	PCR	47	43	4	0	0	0	0
88	Tanimoto et al., 2010 [105]	Vietnam	5’UTR/core, NS5B	phylogeny	114	75	1	10	0	0	28
89	Tokita et al., 1994 [106] ^#^	Vietnam	–	PCR, phylogeny	47	43	4	0	0	0	0
90	Tran et al., 2003 [107]	Vietnam	5’UTR	PCR	21	9	8	0	1	0	3

GT, genotype; LiPA, line probe assay; RFLP, restriction-fragment length polymorphism; RHA, reverse hybridization assay; Multi-country, more than one Southeast Asian country.

*Study did not present data on HCV subtypes or the data could not be extracted.

^#^Study reports cases of mixed genotype and/or subtypes.

### Pooled prevalence of HCV genotypes in Southeast Asia

**Fig 2:**
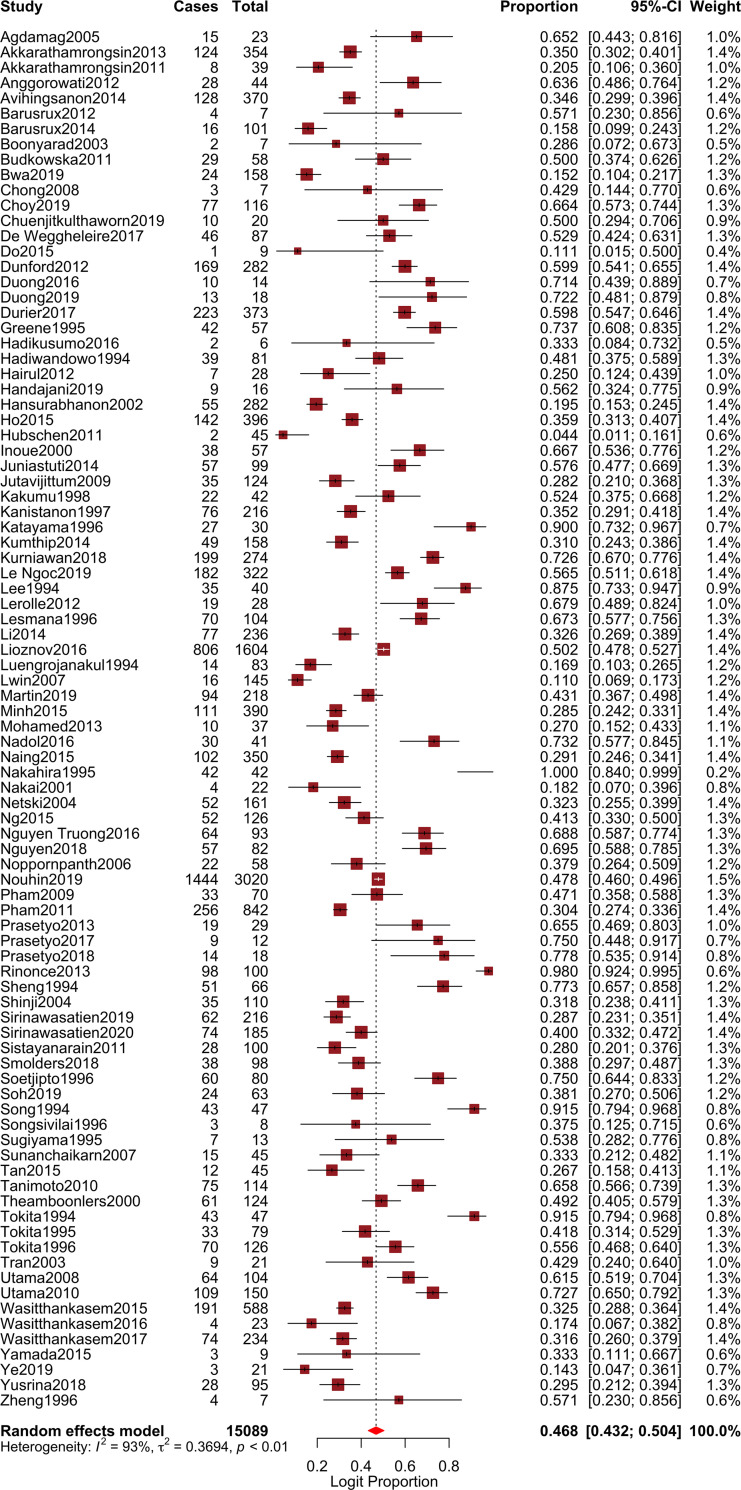
Forest plot showing pooled prevalence of HCV genotype 1 in Southeast Asia.

**Fig 3:**
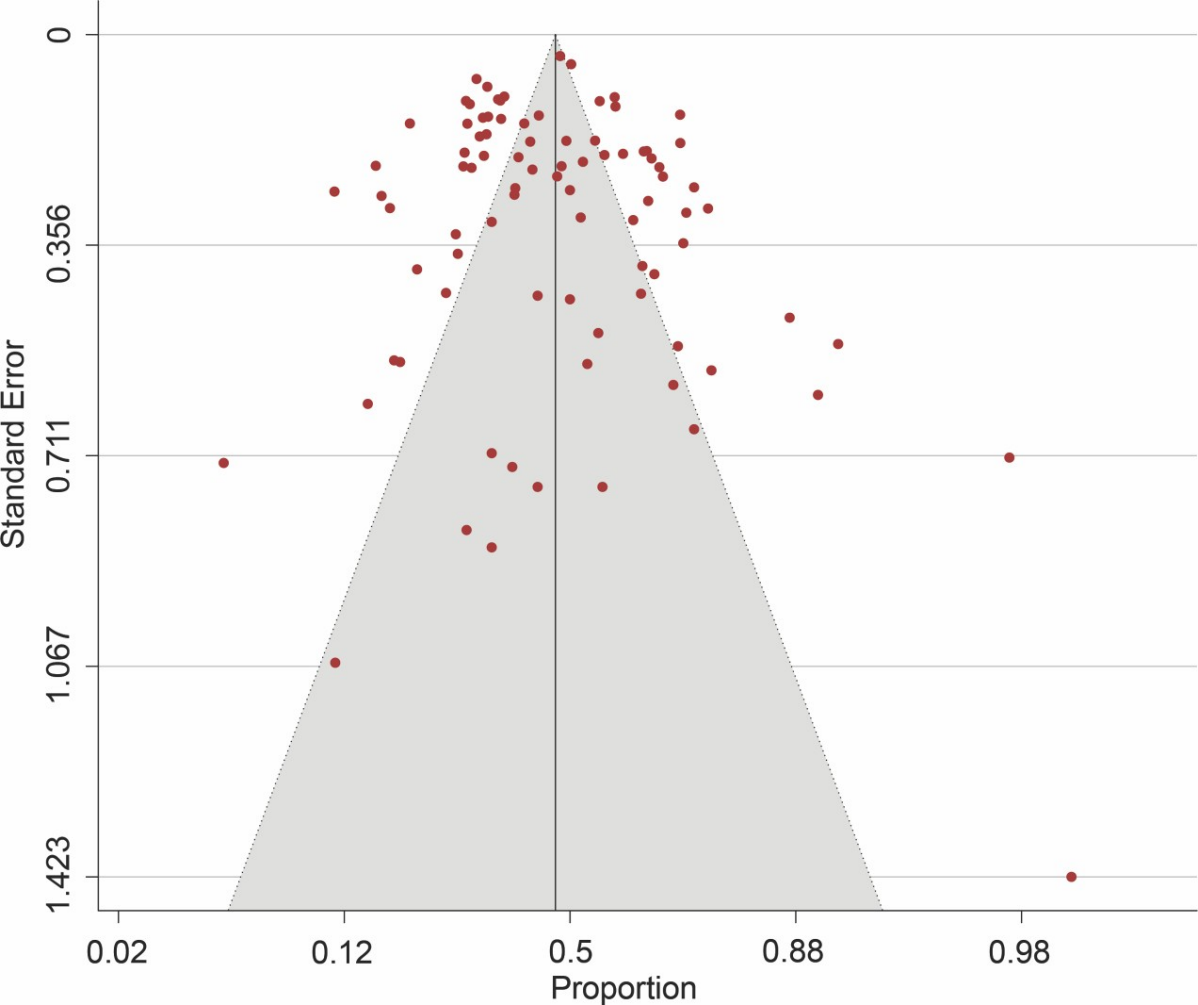
Funnel plot showing no publication bias for the studies reporting HCV genotype 1 in Southeast Asia.

The pooled prevalence of genotype 1 (GT1) was estimated as 46.8% (95% CI, 43.2–50.4; *I*^*2*^ = 92.77%; *p* < 0.001) (**Fig 2**), genotype 2 (GT2) as 4.6% (95% CI, 3.5–5.9; *I*^*2*^ = 88.84%; *p* < 0.001), genotype 3 (GT3) as 23.1% (95% CI, 19.4–27.2; *I*^*2*^ = 93.03%; *p* < 0.001), genotype 4 (GT4) as 1.1% (95% CI, 0.7–1.5; *I*^*2*^ = 49.88%; *p* < 0.001), genotype 5 (GT5) as 0.8% (95% CI, 0.4–1.3; *I*^*2*^ = 79.25%; *p* < 0.001), and genotype 6 (GT6) as 16.5% (95% CI, 13.8–19.6; *I*^*2*^ = 93.98%; *p* < 0.001). Corresponding forest plots are shown in **S1–S5 Figs**. Except for genotype 4 with a moderate heterogeneity, the observed *I*^*2*^ statistics showed high heterogeneity (*I*^*2*^ > 75%) among studies reporting other HCV genotypes. Funnel plot for studies reporting the prevalence of HCV genotype 1 in Southeast Asia showed no publication bias (**Fig 3**) and Egger’s regression test revealed a non-significant *p* value (*p* = 0.495). However, there was evidence of publication bias in studies reporting other genotypes (Egger’s *p* < 0.0001) **(S6–S10 Figs)**. Sensitivity assessment using the leave-one-out analysis did not reveal major changes to the estimate derived for all the HCV genotypes.

### Subgroup meta-analysis

Subgroup analysis was conducted to assess genotype distribution across the Southeast Asian countries and to identify possible source of heterogeneity among the studies. The result of subgroup meta-analysis by the distribution of HCV genotypes across countries revealed different degrees of variability in the studies (**Table 2 and S11–S16 Figs**). Overall, high genotype prevalence estimates were observed for genotypes 1, 3, and 6. For genotype 1, Philippines had the highest estimate (79.5%) although with fewer studies (n = 2) while Laos had the lowest (4.4%) with one study (**Table 2**). Studies from Malaysia and Cambodia showed low heterogeneity (*I*^*2*^ = 30.11% and 31.91%, respectively). For genotype 3, Malaysia had the highest estimate (63.1%) while Cambodia had the lowest (0.7%) (**Table 2**). Also, studies from Malaysia had low heterogeneity (*I*^*2*^ = 27.85%). For genotype 6, the highest estimate (95.6%) was from Laos, although with a single study, meanwhile, Indonesia and Malaysia had the lowest estimates (1.3% each). With the exception of Philippines and Malaysia, heterogeneity was of moderate to high in studies from other countries (**Table 2**).

**Table 2:** Subgroup analysis for comparison of genotype distribution across Southeast Asian countries.

Country	Number of studies	Prevalence (%)	95% CI	*I*^*2*^ (%)	*Q*	Heterogeneity test	
						DF	*p*
** Genotype 1**							
Philippines	2	79.5	45.6–94.7	77.16	4.378	1	0.036
Thailand	27	32.9	29.5–36.4	75.79	107.412	26	< 0.001
Indonesia	17	62.7	61.4–72.6	73.77	61.005	16	< 0.001
Cambodia	5	50.2	44.6–55.8	31.91	5.875	4	0.209
Myanmar	6	19.8	13.2–28.7	82.83	29.114	5	< 0.001
Brunei	1	42.9	14.4–77.0	–	–	–	–
Singapore	3	65.7	38.3–85.5	91.71	24.122	2	< 0.001
Vietnam	19	56.1	48.3–63.6	94.23	312.136	18	< 0.001
Multi-country*	3	54.3	31.8–72.5	94.03	33.523	2	< 0.001
Malaysia	6	34.9	29.5–40.7	30.11	7.154	5	0.209
Laos	1	4.4	1.1–16.1	–	–	–	–
Overall	90	46.8	43.2–50.4	92.77	1230.186	89	< 0.001
** Genotype 2 **							
Philippines	2	20.5	5.3–54.4	77.16	4.378	1	0.036
Thailand	27	1.1	0.5–2.6	82.46	148.233	26	< 0.001
Indonesia	17	16.4	11.7–22.5	78.55	74.602	16	< 0.001
Cambodia	5	4.4	3.8–5.2	0	1.417	4	0.841
Myanmar	6	1.0	0.5–2.1	0	1.851	5	0.869
Brunei	1	14.3	2.0–58.1	–	–	–	–
Singapore	3	2.2	0.6–7.8	28.18	2.785	2	0.248
Vietnam	19	10.6	7.9–14.0	80.40	91.830	18	< 0.001
Multi-country*	3	2.3	0.6–8.6	74.12	7.729	2	0.021
Malaysia	6	1.7	0.9–3.1	0	2.081	5	0.838
Laos	1	1.1	0.1–15.1	–	–	–	–
Overall	90	4.6	3.5–5.9	88.84	797.228	89	< 0.001
** Genotype 3 **							
Philippines	2	1.8	0.3–11.9	0	0.017	1	0.897
Thailand	27	45.8	40.7–51.0	87.40	206.310	26	< 0.001
Indonesia	17	13.0	8.4–19.6	83.56	97.306	16	< 0.001
Cambodia	5	0.7	0.1–4.1	58.44	9.625	4	0.047
Myanmar	6	59.0	42.2–55.9	64.89	14.242	5	0.014
Brunei	1	42.9	14.4–77.0	–	–	–	–
Singapore	3	27.7	14.3–46.8	82.86	11.670	2	0.003
Vietnam	19	2.1	1.2–3.7	64.27	50.375	18	< 0.001
Multi-country*	3	30.3	20.0–43.1	82.80	11.630	2	0.003
Malaysia	6	63.1	57.4–68.5	27.85	6.930	5	0.226
Laos	1	1.1	0.1–15.1	–	–	–	–
Overall	90	23.1	19.4–27.2	93.03	1277.648	89	< 0.001
** Genotype 4 **							
Philippines	2	1.8	0.3–11.9	0	0.017	1	0.897
Thailand	27	0.6	0.3–1.0	0	19.590	26	0.811
Indonesia	17	2.7	1.7–4.4	21.44	20.366	16	0.204
Cambodia	5	1.2	0.1–1.86	88.69	35.374	4	< 0.001
Myanmar	6	0.6	0.2–1.7	0	3.158	5	0.676
Brunei	1	6.3	0.4–53.9	–	–	–	–
Singapore	3	2.2	0.4–11.8	52.26	4.189	2	0.123
Vietnam	19	0.6	0.3–1.2	11.77	20.402	18	0.311
Multi-country*	3	1.9	1.0–3.6	0	1.288	2	0.525
Malaysia	6	1.2	0.4–3.8	13.00	5.747	5	0.332
Laos	1	1.1	0.1–15.1	–	–	–	–
Overall	90	1.1	0.7–1.5	49.88	177.585	89	< 0.001
** Genotype 5 **							
Philippines	2	1.8	0.3–11.9	0	0.017	1	0.897
Thailand	27	0.8	0.2–2.8	89.12	238.885	26	< 0.001
Indonesia	17	0.9	0.5–1.8	0	7.755	16	0.956
Cambodia	5	0.6	0.1–3.8	57.04	9.310	4	0.054
Myanmar	6	0.6	0.2–1.7	0	3.158	5	0.676
Brunei	1	6.3	0.4–53.9	–	–	–	–
Singapore	3	0.7	0.1–3.6	0	0.279	2	0.870
Vietnam	19	0.6	0.3–1.0	0	17.021	18	0.522
Multi-country*	3	0.4	0.1–1.9	0	0.933	2	0.627
Malaysia	6	0.9	0.3–2.9	0	4.451	5	0.486
Laos	1	1.1	0.1–15.1	–	–	–	–
Overall	90	0.8	0.4–1.3	79.25	428.906	89	< 0.001
** Genotype 6 **							
Philippines	2	1.8	0.3–11.9	0	0.017	1	0.897
Thailand	27	16.7	13.4–20.7	85.19	175.546	26	< 0.001
Indonesia	17	1.3	0.6–3.1	57.39	37.553	16	0.002
Cambodia	5	45.7	36.7–54.9	61.09	10.280	4	0.036
Myanmar	6	29.0	18.1–43.0	90.90	54.930	5	< 0.001
Brunei	1	6.3	0.4–59.3	–	–	–	–
Singapore	3	2.5	0.3–20.4	72.23	7.203	2	0.027
Vietnam	19	34.6	28.0–41.8	92.96	255.582	18	< 0.001
Multi-country*	3	12.8	5.8–26.0	82.11	11.179	2	0.004
Malaysia	6	1.3	0.6–2.9	0	4.165	5	0.526
Laos	1	95.6	83.9–98.9	–	–	–	–
Overall	90	16.5	13.8–19.6	93.98	1479.448	89	< 0.001

*Genotyped and analyzed samples from more than one Southeast Asian country.

Subgroup meta-analysis based on the period of data collection also revealed different degrees of variability in the studies (**Table 3**). Before year 2000, the prevalence estimates showed that genotype 1 (60.1%; 95% CI, 49.8–69.6; *I*^*2*^ = 91.50%) was the most prevalent HCV genotype followed by genotype 3 (15.5%; 95% CI, 9.8–23.6; *I*^*2*^ = 89.10%) (**Table 3**). Although with a reduced prevalence, the pooled estimates for year 2000–2009 also revealed genotype 1 (40.7%; 95% CI, 33.2–48.6; *I*^*2*^ = 93.05%) as the most dominant genotype. The estimate for genotype 3 (21.3%; 95% CI, 13.8–31.1; *I*^*2*^ = 94.52%) on the other hand was higher than the earlier decade. A high prevalence (20.5%; 95% CI, 13.9–29.2; *I*^*2*^ = 94.48%) was also observed for genotype 6 during the 2000–2009 study period. For the period of 2010–2020, the prevalence of genotypes 1 and 3 increased to 44.5% (95% CI, 40.1–49.0; *I*^*2*^ = 92.95) and 27.0% (95% CI, 21.9–32.9; *I*^*2*^ = 93.38) respectively, while genotype 6 decreased to 19.6% (95% CI, 15.8–24.1; *I*^*2*^ = 94.95). Except for genotype 4 and the periods between 2000–2020 of genotype 5, heterogeneity was generally high (*I*^*2*^ > 75%) in the study periods under consideration.

**Table 3:** Subgroup analysis for comparison of genotype distribution in Southeast Asia based on the period of data collection.

Period of data collection	Number of studies	Prevalence (%)	95% CI	*I*^*2*^ (%)	*Q*	Heterogeneity test	
						DF	*p*
** Genotype 1**							
< 2000	22	60.1	49.8–69.6	91.50	246.973	21	< 0.001
2000–2009	24	40.7	33.2–48.6	93.05	330.765	23	< 0.001
2010–2020	44	44.5	40.1–49.0	92.95	609.605	43	< 0.001
Overall	90	46.8	43.2–50.4	92.77	1230.186	89	< 0.001
** Genotype 2 **							
< 2000	22	12.1	8.3–17.2	78.06	95.724	21	< 0.001
2000–2009	24	4.2	2.4–7.0	82.94	134.849	23	< 0.001
2010–2020	44	2.5	1.7–3.8	90.99	477.014	43	< 0.001
Overall	90	4.6	3.5–5.9	88.84	797.228	89	< 0.001
** Genotype 3 **							
< 2000	22	15.5	9.8–23.6	89.10	192.586	21	< 0.001
2000–2009	24	21.2	13.8–31.1	94.52	419.506	23	< 0.001
2010–2020	44	27.0	21.9–32.9	93.38	649.669	43	< 0.001
Overall	90	23.1	19.4–27.2	93.03	1277.648	89	< 0.001
** Genotype 4 **							
< 2000	22	1.2	0.7–2.0	0	9.890	21	0.980
2000–2009	24	1.0	0.4–2.4	67.89	71.628	23	< 0.001
2010–2020	44	1.0	0.6–1.6	52.78	91.073	43	< 0.00
Overall	90	1.1	0.7–1.5	49.88	177.585	89	< 0.00
** Genotype 5 **							
< 2000	22	1.3	0.4–4.7	87.06	162.244	21	< 0.001
2000–2009	24	0.7	0.4–1.2	0	14.872	23	0.899
2010–2020	44	0.6	0.4–0.9	0	43.161	43	0.464
Overall	90	0.8	0.4–1.3	79.25	428.906	89	< 0.001
** Genotype 6 **							
< 2000	22	4.0	2.2–7.2	80.86	109.699	21	< 0.001
2000–2009	24	20.5	13.9–29.2	94.48	416.986	23	< 0.001
2010–2020	44	19.6	15.8–24.1	94.95	852.257	43	< 0.001
Overall	90	16.5	13.8–19.6	93.98	1479.449	89	< 0.001

### Distribution of HCV subtypes in Southeast Asia

In this study, a total of 9,646 HCV subtypes were reported across the 69 studies and ranged from 3 (in Malaysia) to 2,950 (in Cambodia). Overall, data on HCV subtypes were available in studies from Brunei (n = 1), Cambodia (n = 4), Indonesia (n = 15), Laos (n = 1), Malaysia (n = 3), Myanmar (n = 4), Philippines (n = 2), Singapore (n = 1), Thailand (n = 21), and Vietnam (n = 14). Three other studies that contributed to the subtype data were studies categorized as “multi-country” (**Table 1 and S3 File**). The following subtypes were reported: subtype of genotype 1 (1a-1e), 2 (2a-2c, 2e, 2f, 2i-2k, and 2m), 3 (3a-3c, 3g, 3k), 4 (4a), and 6 (6a-6c, 6e, 6f, 6h-6v, 6xa-6xc, 6xf) (**Table 4**).

**Table 4:** Prevalence of HCV subtypes in Southeast Asia.

HCV subtype	Pooled prevalence (%)	95% CI
**1a**	21.3	17.0–26.4
**1b**	26.3	22.1–30.9
**1c**	2.0	1.3–3.1
**1d**	0.9	0.6–1.3
**1e**	0.9	0.7–1.2
**2a**	3.4	2.4–4.8
**2b**	1.1	0.7–1.8
**2c**	1.1	0.7–1.5
**2e**	1.2	0.9–1.7
**2f**	1.0	0.7–1.3
**2i**	1.1	0.8–1.5
**2j**	0.9	0.6–1.2
**2k**	0.9	0.6–1.2
**2m**	1.1	0.8–1.5
**3a**	14.3	11.0–18.5
**3b**	3.6	2.5–5.1
**3c**	0.9	0.7–1.3
**3g**	0.9	0.7–1.3
**3k**	1.4	0.9–2.3
**4a**	1.2	0.8–1.6
**6a**	2.8	1.9–4.1
**6b**	1.1	0.8–1.6
**6c**	0.9	0.7–1.2
**6e**	2.6	1.8–3.6
**6f**	1.8	1.2–2.7
**6h**	1.1	0.8–1.6
**6i**	1.5	1.1–2.0
**6j**	1.1	0.8–1.5
**6k**	0.9	0.7–1.3
**6l**	1.3	0.9–1.8
**6m**	1.1	0.7–1.6
**6n**	1.6	1.0–2.5
**6o**	1.0	0.7–1.2
**6p**	1.7	1.4–2.1
**6q**	1.1	0.8–1.6
**6r**	1.1	0.7–1.8
**6s**	1.6	1.3–1.9
**6t**	0.9	0.6–1.2
**6u**	0.8	0.6–1.1
**6v**	0.9	0.6–1.2
**6xa**	1.0	0.7–1.5
**6xb**	0.9	0.6–1.2
**6xc**	0.9	0.6–1.2
**6xf**	1.6	1.3–2.0

The three most prevalent HCV subtypes in the Southeast Asian region were 1b (26.3%; 95% CI, 22.1–30.9), 1a (21.3%; 95% CI, 17.0–26.4), and 3a (14.3%; 95% CI, 11.0–18.5). The prevalence of other reported subtypes was less than 4% (**Table 4)**.

Mixed genotypes and/or subtypes were identified in reports from some of the countries. They include Indonesia [(1b+2b+3b), (1a+1b), and (3a+3b)], Malaysia [(1+3), (3+4) and (1a+1b)], Thailand [(1+2), (1+3), (1+4), (1+5), (2+5), (3+6), (1+3+4), (1a+1b), (1a+3b), (1b+3a), (3a+3b) and (3a+6)], Vietnam [(1a+1b), (1a+2b), (1b+2a), (1a+1b+2b) and (1a+2b+3a)], and Multi-country (1a+3) (**Table 1**).

## Discussion

The genotypic diversity of HCV has for several years been a major hurdle to drug and vaccine development. While there are effective antiviral drugs against HCV, there is yet to be a licensed vaccine. Thus, antiviral therapy remains the mainstay of managing HCV infection. Numerous studies have demonstrated that different antiviral compounds and drugs (including the more recent interferon-free direct-acting antiviral agents such as telaprevir, sofosbuvir, etc.) display variable antiviral activities against the infecting genotypes and subtypes of HCV [108–114], a concern largely attributed to resistance associated substitutions in HCV [115, 116]. A clear knowledge of the actual distribution of HCV genotype and subtypes in a region is pivotal not only to the treatment of the infection and the potential selection of candidate genotypic vaccine target for the region, but also to the development of appropriate government policies, interventions and programs.

In this study, we present a detailed and comprehensive view of HCV genotype distribution in SEA from available published regional data since the discovery of the virus in 1989. Apart from East Timor, genotype data was available for all the countries in this region. Most of the studies were from Thailand, Vietnam and Indonesia; possibly because of the burden of HCV infection [117–119] in relation to their high population. Similar to many other nations across the globe, we identified genotype 1 as the most dominant genotype in SEA. While this finding does not contradict the popular notion that genotype 6 is prevalent in SEA as supported by the extensive global study conducted by Messina et al. [4], it underscores the need for a cautious interpretation of results of studies conducted at global and regional levels. It is pertinent to clarify that genotype 6 is more prevalent in SEA than any other region in the world when discussed according to global prevalence. However, within SEA, genotype 1 is the most prevalent, followed by genotypes 3 and 6, respectively (**Table 2**). Combination of the pooled prevalence of these genotypes (1,3 and 6) accounted for more than 85% of the genotypes reported in the region, indicating that the three genotypes are the major HCV genotypes of concern. Notably, we found that the highest prevalence for genotype 1 (79.5%; 95% CI, 45.6–94.7), and genotype 2 (20.5; 95% CI, 5.3–54.4) occurred in Philippines and with an even distribution of the other genotypes. In addition, we observed that genotype 6 became more prevalent from the first decade of the twenty-first century. As suggested by previous reports [4, 118], our study showed that genotypes 4 and 5 were almost inexistent in SEA as only few isolated cases have been reported.

Although the prevalence of HCV subtypes appears to vary in this study, we identified more than forty different subtypes (**Table 3**), indicating the presence of a marked diversity of the virus’ subtypes in the region. Overall, subtypes 1b (26.3%; 95% CI, 22.1–30.9), 1a (21.3%; 95% CI, 17.0–26.4), and 3a (14.3%; 95% CI, 11.0–18.5) constitute the major subtypes in SEA identified by this study. The predominance of subtype 1b corroborates earlier reports on the subtype as the predominant HCV subtype across the globe [4, 11]. In addition to the cases of dual ‘mixed’ genotype infections identified in this study, several cases involving a mixture of more than two genotypes and/or subtypes in patients were reported in Indonesia, Vietnam, and Thailand [28, 80, 93]. Expectedly, such infected individuals would require more than the usual combinations of the available HCV drugs for optimal treatment outcomes. Given the high cost of treatment, the occurrence of ‘mixed’ genotypes and subtypes revealed in this study unveils an additional hurdle that should be considered in the current race for a vaccine, as agents capable of generating cross-reactive immunity would be highly invaluable.

This study however has its limitations. There was paucity of studies in some of the included SEA countries. For example, the estimates for Brunei and Laos were derived from one study each. This could amount to an underestimation of genotypes and subtypes. Another concern is the variations in the methods of genotyping, as well as the targeted region for genotyping. Furthermore, because we prioritized peer-reviewed publications in an attempt to limit the tendency of including studies with unreliable genotype reports, we could have missed some relevant studies. Nevertheless, a broad search of multiple databases was done to access as many studies as possible in order to curtail the impact of overlooked studies. Despite these limitations, we believe this study provides a comprehensive and up-to-date situation of HCV genotype distribution in SEA.

## Supporting information

S1 Fig Forest plot showing pooled prevalence of HCV genotype 2 in Southeast Asia. (PDF)

S2 Fig Forest plot showing pooled prevalence of HCV genotype 3 in Southeast Asia. (PDF)

S3 Fig Forest plot showing pooled prevalence of HCV genotype 4 in Southeast Asia. (PDF)

S4 Fig Forest plot showing pooled prevalence of HCV genotype 5 in Southeast Asia. (PDF)

S5 Fig Forest plot showing pooled prevalence of HCV genotype 6 in Southeast Asia. (PDF)

S6 Fig Funnel plot of studies reporting HCV genotype 2 in Southeast Asia. (EPS)

S7 Fig Funnel plot of studies reporting HCV genotype 3 in Southeast Asia. (EPS)

S8 Fig Funnel plot of studies reporting HCV genotype 4 in Southeast Asia. (EPS)

S9 Fig Funnel plot of studies reporting HCV genotype 5 in Southeast Asia. (EPS)

S10 Fig Funnel plot of studies reporting HCV genotype 6 in Southeast Asia. (EPS)

S11 Fig Forest plot showing pooled prevalence of HCV genotype 1 according to Southeast Asian countries. (PDF)

S12 Fig Forest plot showing pooled prevalence of HCV genotype 2 according to Southeast Asian countries. (PDF)

S13 Fig Forest plot showing pooled prevalence of HCV genotype 3 according to Southeast Asian countries. (PDF)

S14 Fig Forest plot showing pooled prevalence of HCV genotype 4 according to Southeast Asian countries. (PDF)

S15 Fig Forest plot showing pooled prevalence of HCV genotype 5 according to Southeast Asian countries. (PDF)

S16 Fig Forest plot showing pooled prevalence of HCV genotype 6 according to Southeast Asian countries. (PDF)

S1 Table Quality assessment of the included studies. (PDF)

S2 Table PRISMA 2009 checklist. (DOC)

S1 File Search strategy. (PDF)

S2 File Joanna Briggs Institute (JBI) critical appraisal checklist for prevalence data. (PDF)

S3 File Data extraction sheet for HCV subtypes. (XLSX)
