# Supplementary figures and images for: Prevalence of HCV genotypes and subtypes in Southeast Asia: A systematic review and meta-analysis
Source: PLoS One. 2021 May 20;16(5):e0251673. doi: 10.1371/journal.pone.0251673 (PMC8136688; doi:10.1371/journal.pone.0251673)

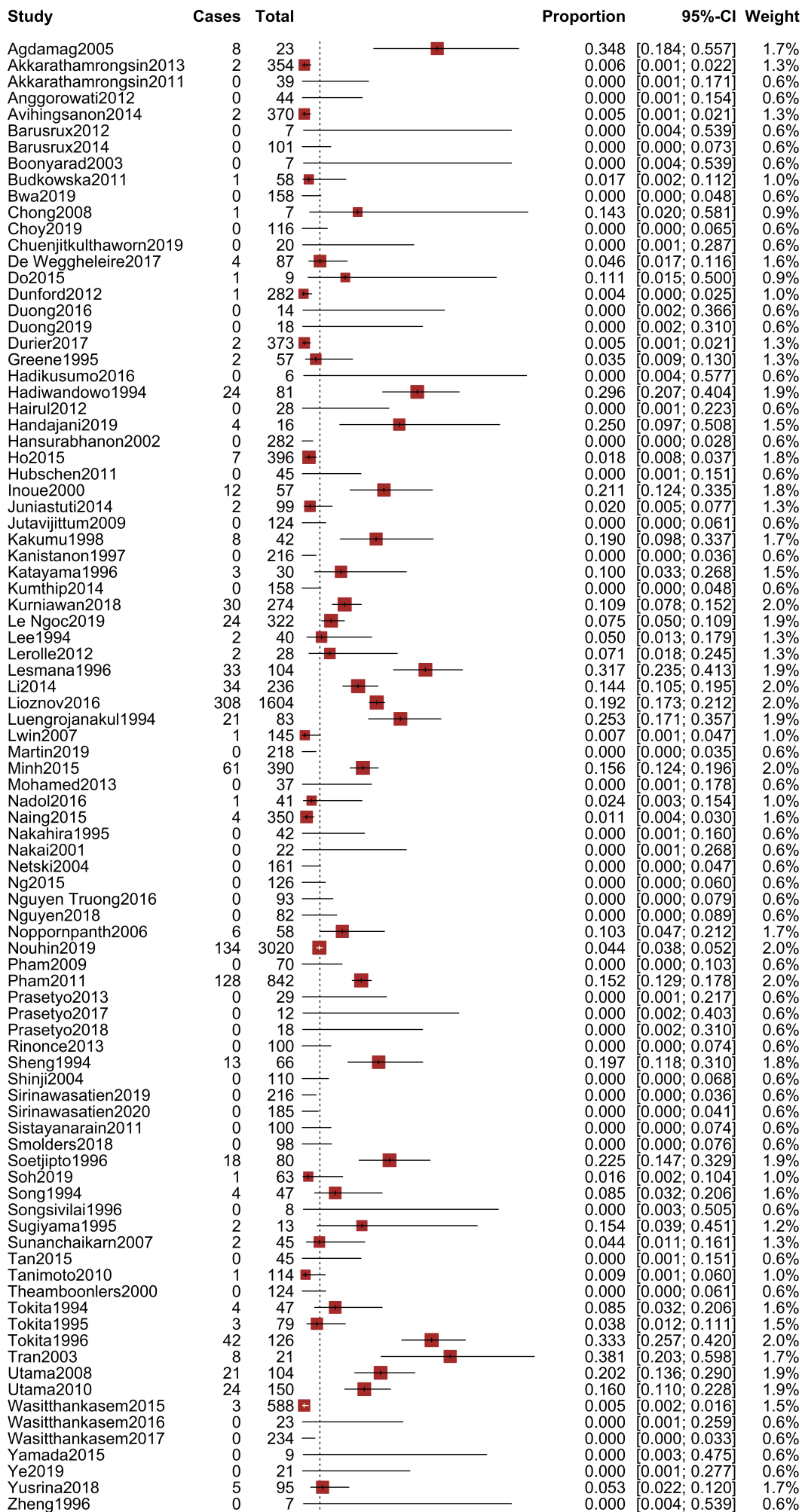

**Random effects model** 15089 **0.046 [0.035; 0.059] 100.0%**

Heterogeneity:  $I^2 = 89\%$ ,  $\tau^2 = 0.9281$ ,  $p < 0.01$

0.1 0.2 0.3 0.4 0.5  
Logit Proportion

Supplement: S1 Fig — (PDF) [file pone.0251673.s001.pdf]

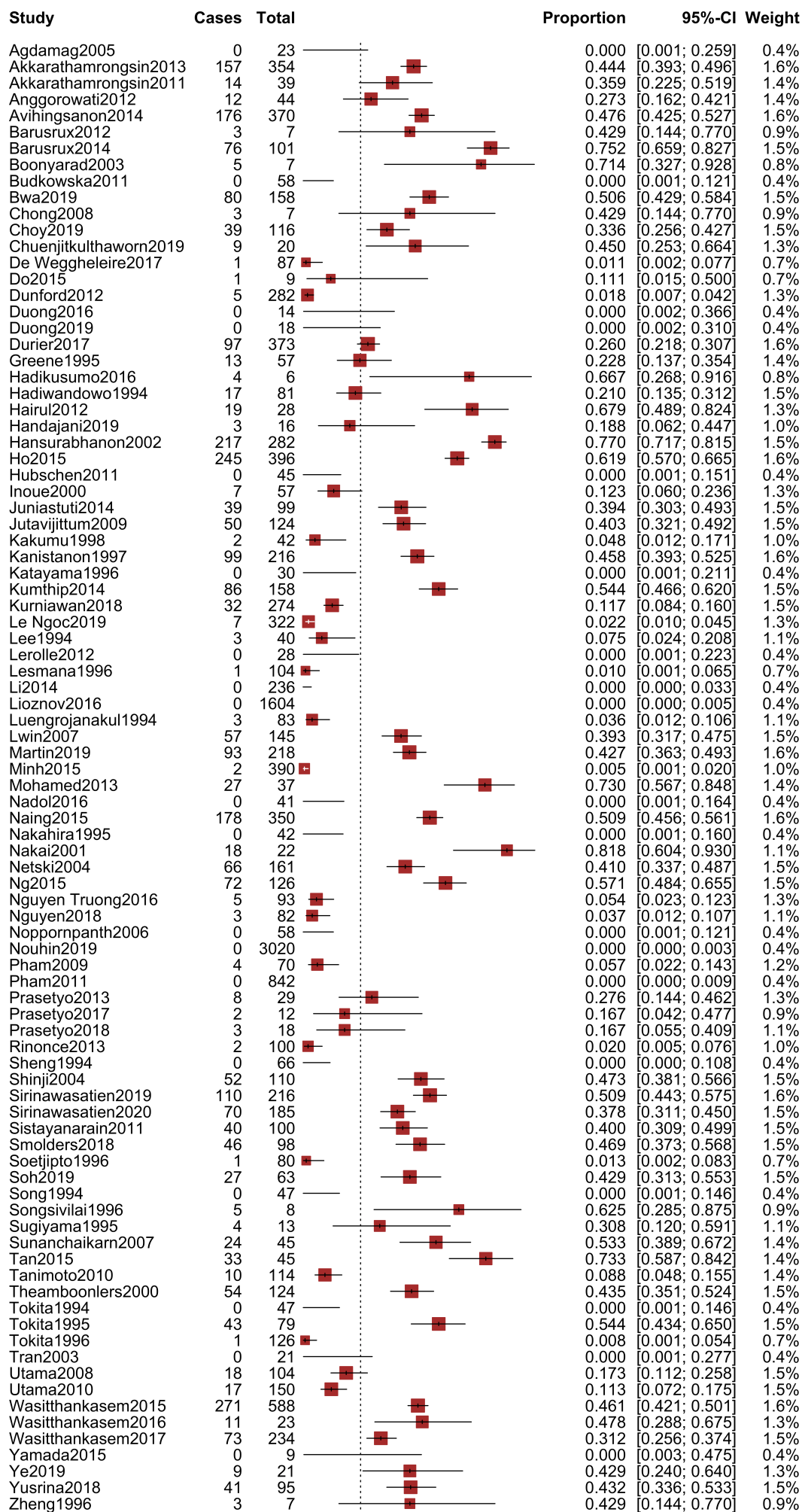

**Random effects model** 15089  
Heterogeneity:  $I^2 = 93\%$ ,  $\tau^2 = 0.7859$ ,  $p < 0.01$

**0.231 [0.194; 0.272] 100.0%**

0.2 0.4 0.6 0.8  
Logit Proportion

Supplement: S2 Fig — (PDF) [file pone.0251673.s002.pdf]

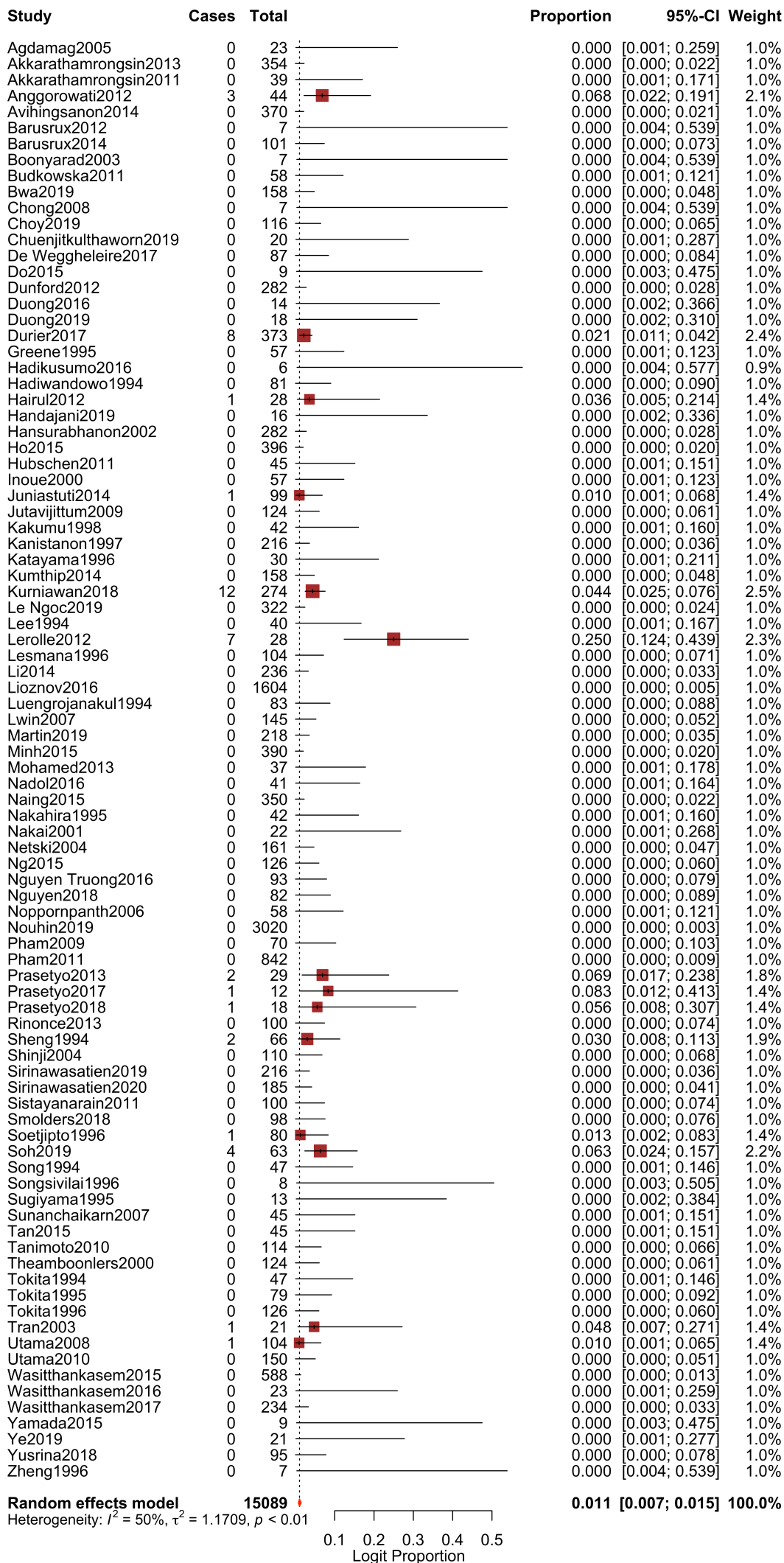

Supplement: S3 Fig — (PDF) [file pone.0251673.s003.pdf]

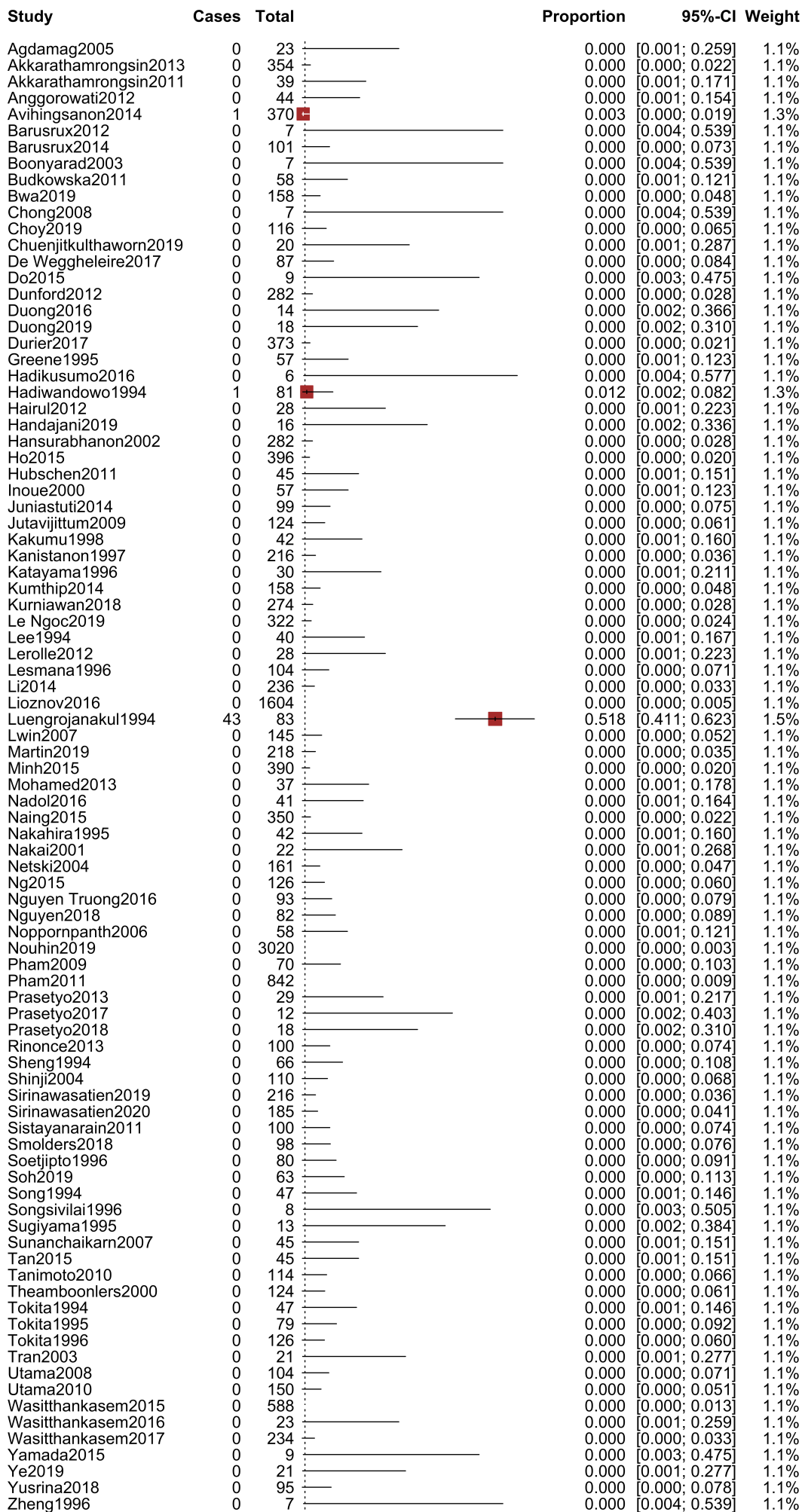

**Random effects model** 15089  
Heterogeneity:  $I^2 = 79\%$ ,  $\tau^2 = 5.7898$ ,  $p < 0.01$   
0.1 0.2 0.3 0.4 0.5 0.6  
Logit Proportion  
**0.008 [0.004; 0.013] 100.0%**

Supplement: S4 Fig — (PDF) [file pone.0251673.s004.pdf]

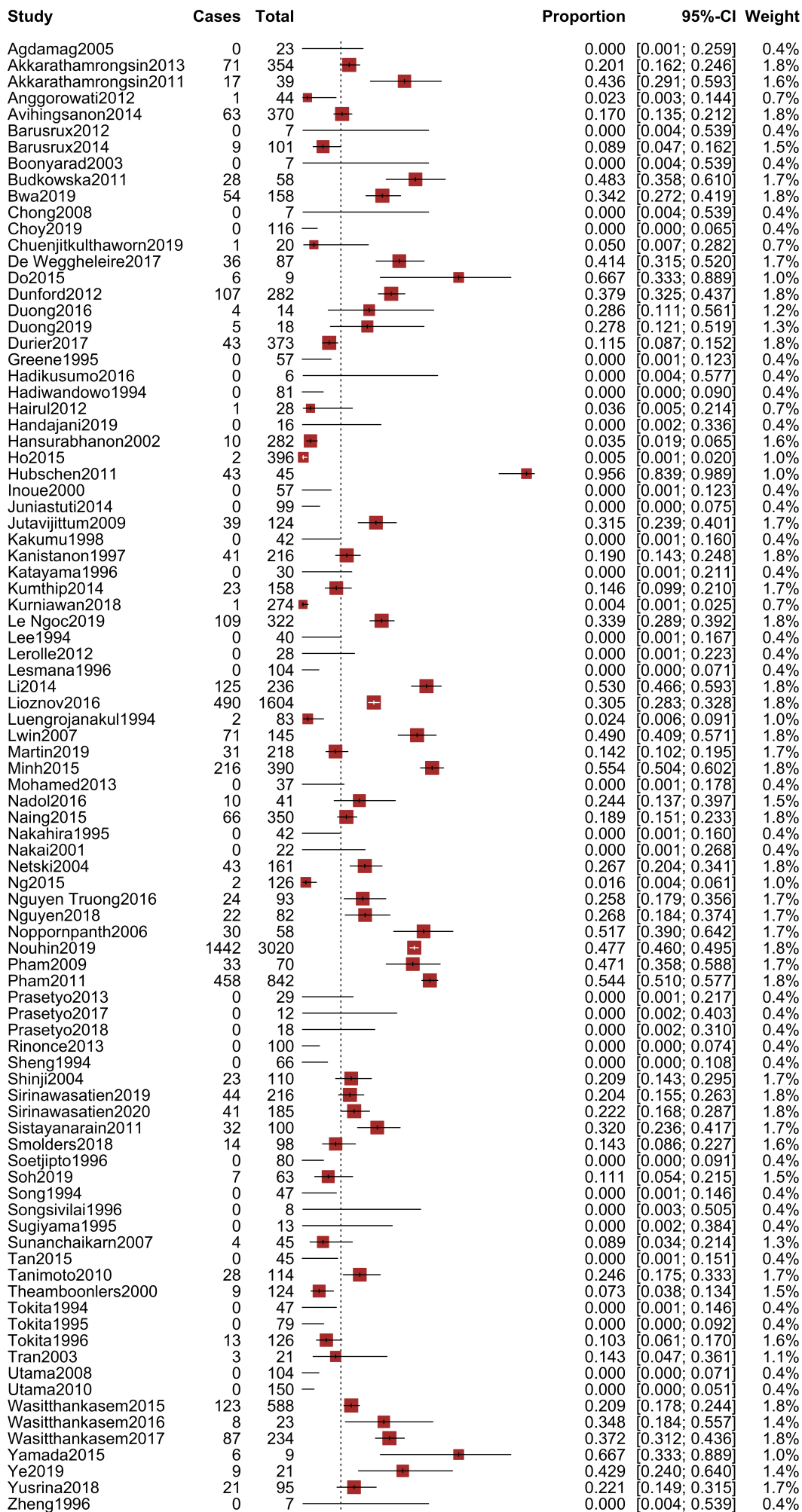

**Random effects model** 15089  
Heterogeneity:  $I^2 = 94\%$ ,  $\tau^2 = 0.6255$ ,  $p < 0.01$

**0.165 [0.138; 0.196] 100.0%**

0.2 0.4 0.6 0.8  
Logit Proportion

Supplement: S5 Fig — (PDF) [file pone.0251673.s005.pdf]

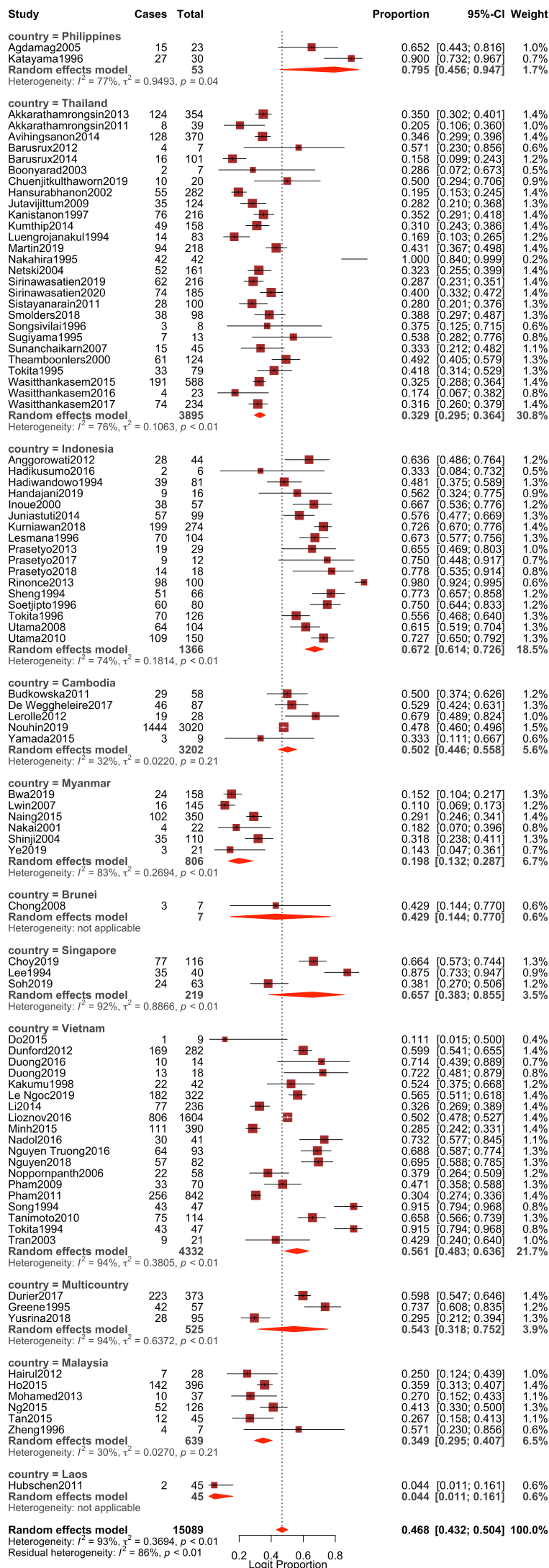

Supplement: S11 Fig — (PDF) [file pone.0251673.s011.pdf]

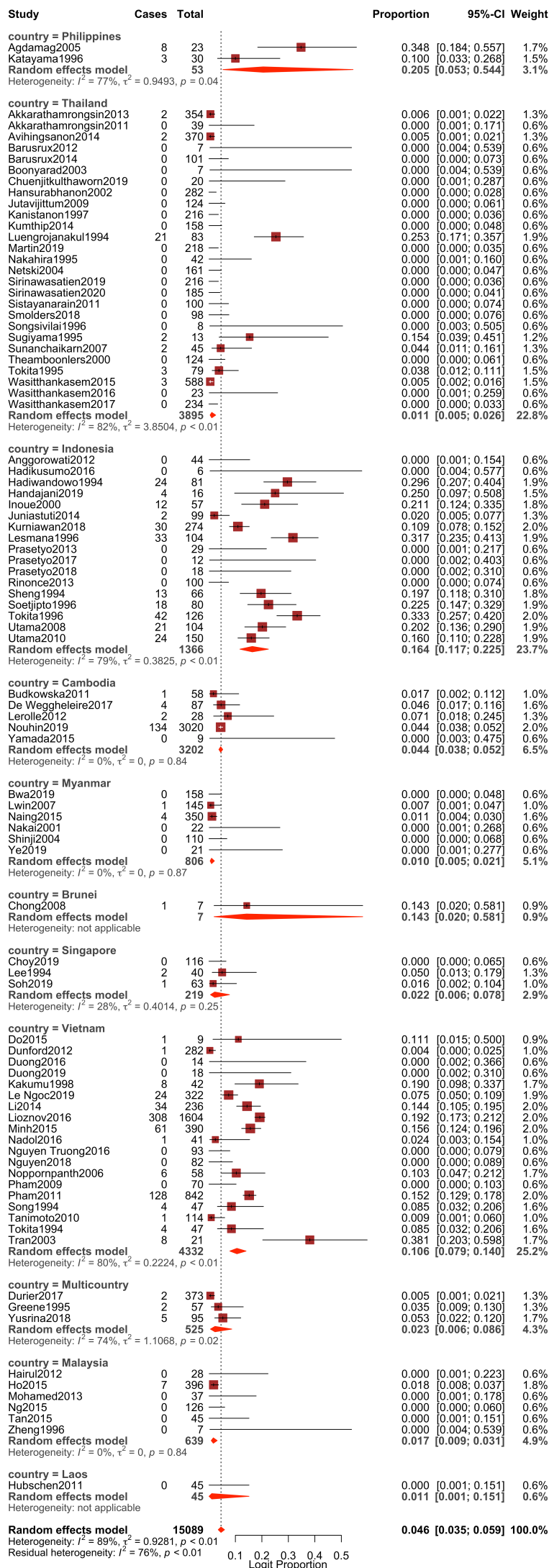

Supplement: S12 Fig — (PDF) [file pone.0251673.s012.pdf]

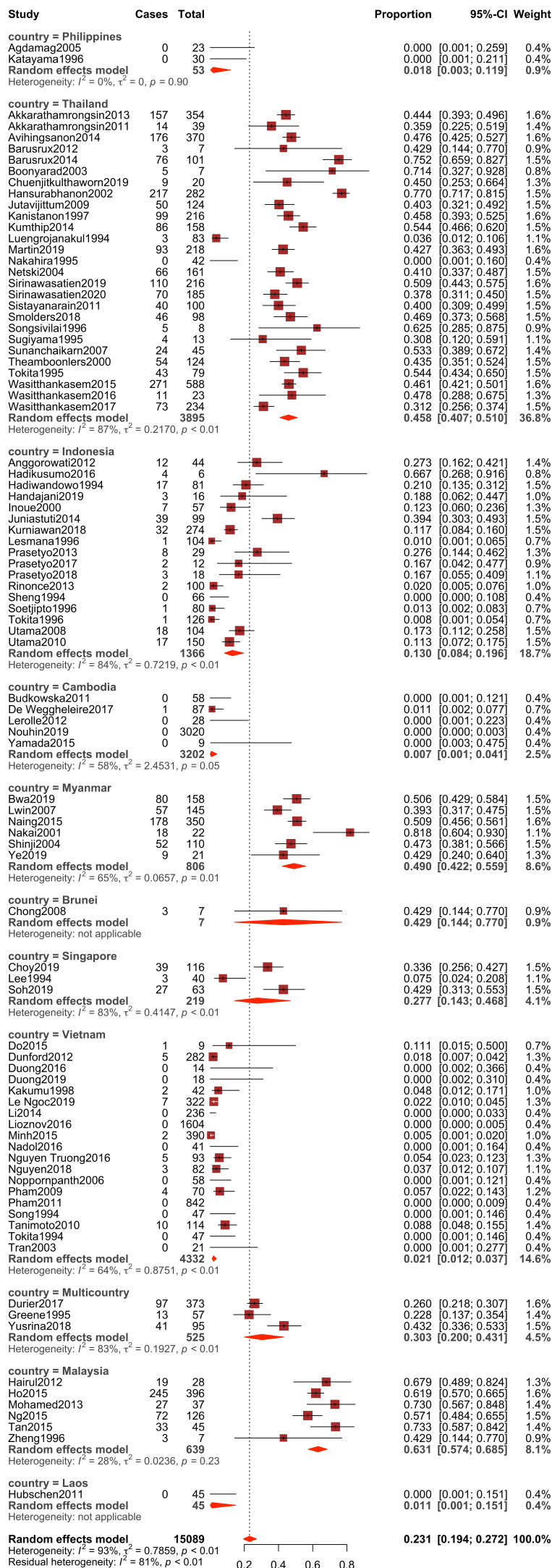

Supplement: S13 Fig — (PDF) [file pone.0251673.s013.pdf]

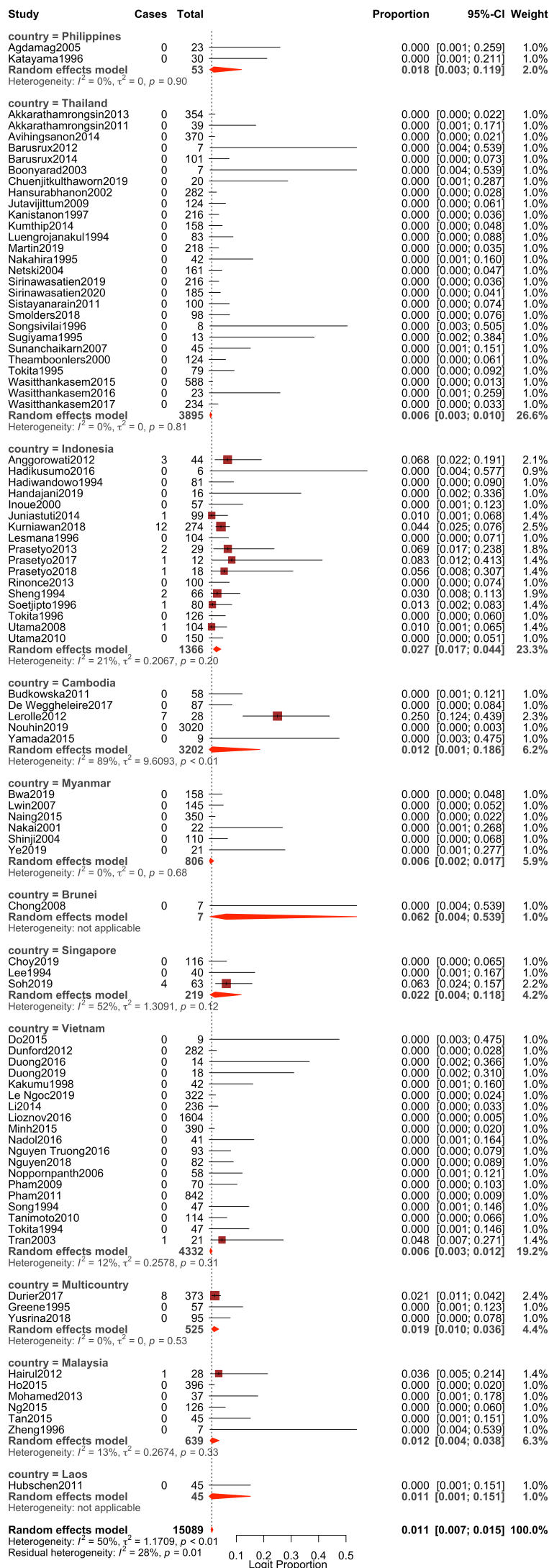

Supplement: S14 Fig — (PDF) [file pone.0251673.s014.pdf]

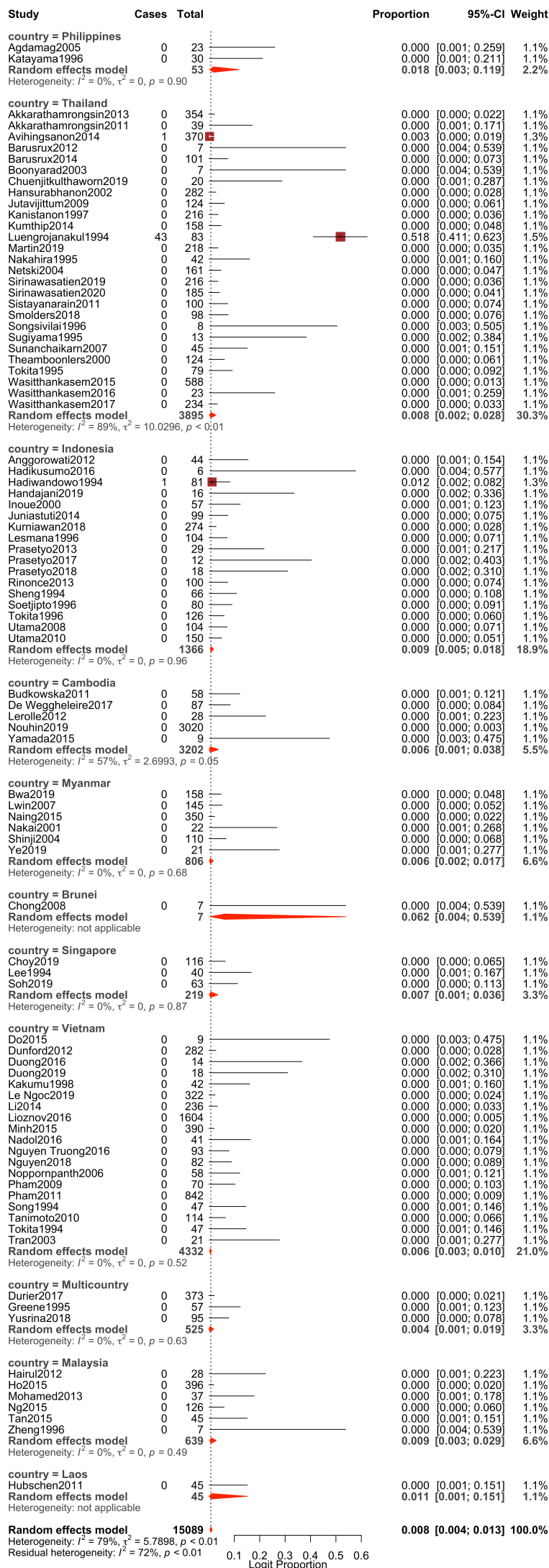

Supplement: S15 Fig — (PDF) [file pone.0251673.s015.pdf]

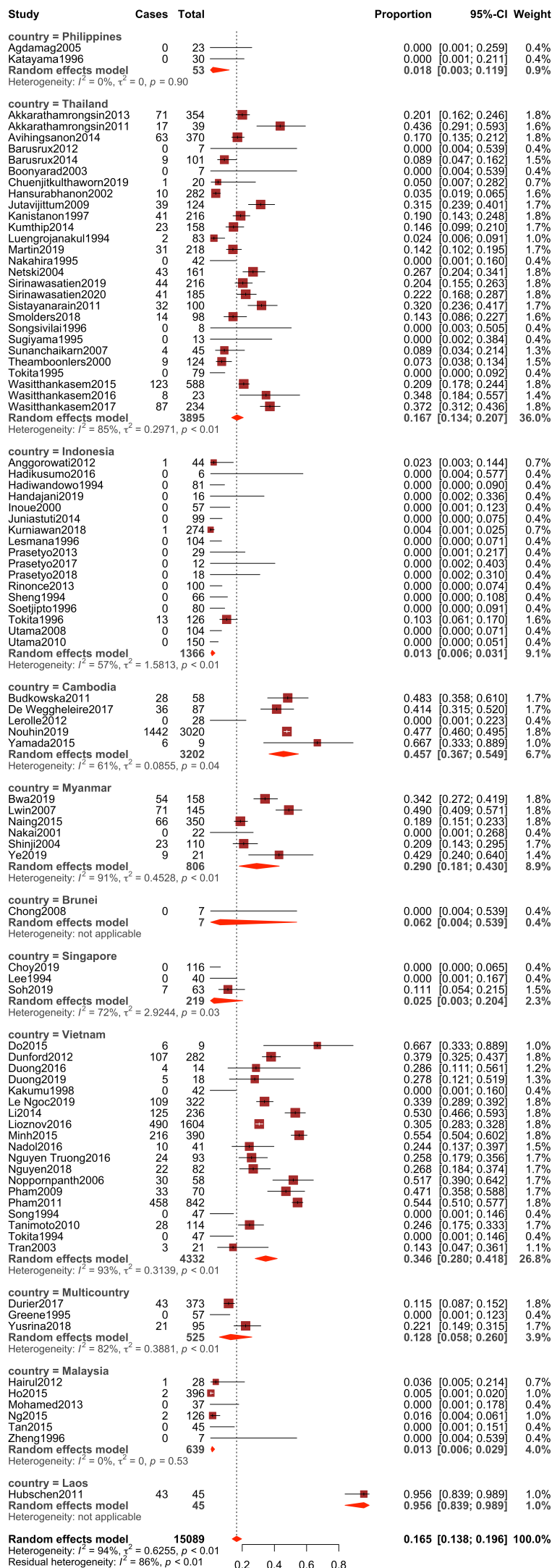

Supplement: S16 Fig — (PDF) [file pone.0251673.s016.pdf]
