# Supplementary material for: Prevalence of HCV genotypes and subtypes in Southeast Asia: A systematic review and meta-analysis
Source: PLoS One. 2021 May 20;16(5):e0251673. doi: 10.1371/journal.pone.0251673 (PMC8136688; doi:10.1371/journal.pone.0251673)
Supplement: S1 Table — (PDF) [file pone.0251673.s017.pdf]

Quality of included studies by JBI critical appraisal checklist for studies reporting prevalence data

Study name		Checklist*									Overall
		1	2	3	4	5	6	7	8	9	
1	Chong et al., 2008	Yes	No	Yes	Yes	Yes	Yes	Yes	Yes	Unclear	7
2	Budkowska et al., 2011	Yes	No	Yes	Yes	Yes	Yes	Yes	Yes	Yes	8
3	De Weggheleire et al., 2017	Yes	No	Yes	Yes	Yes	Yes	Yes	Yes	Yes	8
4	Lerolle et al., 2012	Yes	No	Yes	Yes	Yes	Yes	Yes	Yes	Yes	8
5	Nouhin et al., 2019	Yes	No	Yes	Yes	Yes	Yes	Yes	Yes	Yes	8
6	Yamada et al., 2015	Yes	No	Yes	Yes	Yes	Yes	Yes	Yes	Yes	8
7	Anggorowati et al., 2012	Yes	No	Yes	Yes	Yes	Yes	Yes	Yes	Yes	8
8	Hadikusumo et al., 2016	Yes	No	Yes	Yes	Yes	Yes	Yes	Yes	Yes	8
9	Hadiwandowo et al., 1994	Yes	No	Yes	Yes	Yes	Yes	Yes	Yes	Yes	8
10	Handajani et al., 2019	Yes	No	Yes	Yes	Yes	Yes	Yes	Yes	Yes	8
11	Inoue et al., 2000	Yes	No	Yes	Yes	Yes	Yes	Yes	Yes	Yes	8
12	Juniastuti et al., 2014	Yes	No	Yes	Yes	Yes	Yes	Yes	Yes	Yes	8
13	Kurniawan et al., 2018	Yes	No	Yes	Yes	Yes	Yes	Yes	Yes	Yes	8
14	Lesmana et al., 1996	Yes	No	Yes	Yes	Yes	Yes	Yes	Yes	Yes	8
15	Prasetyo et al., 2013	Yes	No	Yes	Yes	Yes	Yes	Yes	Yes	Yes	8
16	Prasetyo et al., 2017	Yes	No	Yes	Yes	Yes	Yes	Yes	Yes	Yes	8
17	Prasetyo et al., 2018	Yes	No	Yes	Yes	Yes	Yes	Yes	Yes	Yes	8
18	Rinonce et al., 2013	Yes	No	Yes	Yes	Yes	Yes	Yes	Yes	Yes	8
19	Sheng et al., 1994	Yes	No	Yes	Yes	Yes	Yes	Yes	Yes	Yes	8
20	Soetjipto et al., 1996	Yes	No	Yes	Yes	Yes	Yes	Yes	Yes	Yes	8
21	Tokita et al., 1996	Yes	No	Yes	Yes	Yes	Yes	Yes	Yes	Yes	8
22	Utama et al., 2008	Yes	No	Yes	Yes	Yes	Yes	Yes	Yes	Yes	8
23	Utama et al., 2010	Yes	No	Yes	Yes	Yes	Yes	Yes	Yes	Yes	8
24	Hübschen et al., 2011	Yes	No	Yes	Yes	Yes	Yes	Yes	Yes	Yes	8
25	Hairul et al., 2012	Yes	No	Yes	Yes	Yes	Yes	Yes	Yes	Yes	8
26	Ho et al., 2015	Yes	No	Yes	Yes	Yes	Yes	Yes	Yes	Yes	8
27	Mohamed et al., 2013	Yes	No	Yes	Yes	Yes	Yes	Yes	Yes	Yes	8
28	Ng et al., 2015	Yes	No	Yes	Yes	Yes	Yes	Yes	Yes	Yes	8
29	Tan et al., 2015	Yes	No	Yes	Yes	Yes	Yes	Yes	Yes	Yes	8
30	Zheng et al., 1996	Yes	No	Yes	Yes	Yes	Yes	Yes	Yes	Yes	8

31	Bwa et al., 2019	Yes	No	Yes	Yes	Yes	Yes	Yes	Yes	Yes	8
32	Lwin et al., 2007	Yes	No	Yes	Yes	Yes	Yes	Yes	Yes	Yes	8
33	Naing et al., 2015	Yes	No	Yes	Yes	Yes	Yes	Yes	Yes	Yes	8
34	Nakai et al., 2001	Yes	No	Yes	Yes	Yes	Yes	Yes	Yes	Yes	8
35	Shinji et al., 2004	Yes	No	Yes	Yes	Yes	Yes	Yes	Yes	Yes	8
36	Ye et al., 2019	Yes	No	Yes	Yes	Yes	Yes	Yes	Yes	Yes	8
37	Agdamag et al., 2005	Yes	No	Yes	Yes	Yes	Yes	Yes	Yes	Yes	8
38	Katayama et al., 1996	Yes	No	Yes	Yes	Yes	Yes	Yes	Yes	Yes	8
39	Durier et al., 2017	Yes	No	Yes	Yes	Yes	Yes	Yes	Yes	Yes	8
40	Greene et al., 1995	Yes	No	Yes	Yes	Yes	Yes	Yes	Yes	Yes	8
41	Yusrina et al., 2018	Yes	No	Yes	Yes	Yes	Yes	Yes	Yes	Yes	8
42	Choy et al., 2019	Yes	No	Yes	Yes	Yes	Yes	Yes	Yes	Yes	8
43	Lee et al., 1994	Yes	No	Yes	Yes	Yes	Yes	Yes	Yes	Yes	8
44	Soh et al., 2019	Yes	No	Yes	Yes	Yes	Yes	Yes	Yes	Yes	8
45	Akkarathamrongsin et al., 2013	Yes	No	Yes	Yes	Yes	Yes	Yes	Yes	Yes	8
46	Akkarathamrongsin et al., 2011	Yes	No	Yes	Yes	Yes	Yes	Yes	Yes	Yes	8
47	Avihingsanon et al., 2014	Yes	No	Yes	Yes	Yes	Yes	Yes	Yes	Yes	8
48	Barusrux et al., 2012	Yes	No	Yes	Yes	Yes	Yes	Yes	Yes	Yes	8
49	Barusrux et al., 2014	Yes	No	Yes	Yes	Yes	Yes	Yes	Yes	Yes	8
50	Boonyarad et al., 2003	Yes	No	Yes	Yes	Yes	Yes	Yes	Yes	Yes	8
51	Chuenjitkulthaworn et al., 2019	Yes	Yes	Yes	Yes	Yes	Yes	Yes	Yes	Yes	9
52	Hansurabhanon et al., 2002	Yes	No	Yes	Yes	Yes	Yes	Yes	Yes	Yes	8
53	Jutavijittum et al., 2009	Yes	No	Yes	Yes	Yes	Yes	Yes	Yes	Yes	8
54	Kanistanon et al., 1997	Yes	No	Yes	Yes	Yes	Yes	Yes	Yes	Yes	8
55	Kumthip et al., 2014	Yes	No	Yes	Yes	Yes	Yes	Yes	Yes	Yes	8
56	Luengrojanakul et al., 1994	Yes	No	Yes	Yes	Yes	Yes	Yes	Yes	Yes	8
57	Martin et al., 2019	Yes	No	Yes	Yes	Yes	Yes	Yes	Yes	Yes	8
58	Nakahira et al., 1995	Yes	No	Yes	Yes	Yes	Yes	Yes	Yes	Yes	8
59	Netski et al., 2004	Yes	No	Yes	Yes	Yes	Yes	Yes	Yes	Yes	8
60	Sirinawasatien et al., 2019	Yes	No	Yes	Yes	Yes	Yes	Yes	Yes	Yes	8
61	Sirinawasatien et al., 2020	Yes	No	Yes	Yes	Yes	Yes	Yes	Yes	Yes	8
62	Sistayanarain et al., 2011	Yes	No	Yes	Yes	Yes	Yes	Yes	Yes	Yes	8

63	Smolders et al., 2018	Yes	No	Yes	Yes	Yes	Yes	Yes	Yes	Yes	8
64	Songsivilai et al., 1996	Yes	No	Yes	Yes	Yes	Yes	Yes	Yes	Yes	8
65	Sugiyama et al., 1995	Yes	No	Yes	Yes	Yes	Yes	Yes	Yes	Yes	8
66	Sunanchaikarn et al., 2007	Yes	No	Yes	Yes	Yes	Yes	Yes	Yes	Yes	8
67	Theamboonlers et al., 2000	Yes	No	Yes	Yes	Yes	Yes	Yes	Yes	Yes	8
68	Tokita et al., 1995	Yes	No	Yes	Yes	Yes	Yes	Yes	Yes	Yes	8
69	Wasitthankasem et al., 2015	Yes	No	Yes	Yes	Yes	Yes	Yes	Yes	Yes	8
70	Wasitthankasem et al., 2016	Yes	No	Yes	Yes	Yes	Yes	Yes	Yes	Yes	8
71	Wasitthankasem et al., 2017	Yes	No	Yes	Yes	Yes	Yes	Yes	Yes	Yes	8
72	Do et al., 2015	Yes	Yes	Yes	Yes	Yes	Yes	Yes	Yes	Yes	9
73	Dunford et al., 2012	Yes	No	Yes	Yes	Yes	Yes	Yes	Yes	Yes	8
74	Duong et al., 2016	Yes	No	Yes	Yes	Yes	Yes	Yes	Yes	Yes	8
75	Duong et al., 2019	Yes	No	Yes	Yes	Yes	Yes	Yes	Yes	Yes	8
76	Kakumu et al., 1998	Yes	No	Yes	Yes	Yes	Yes	Yes	Yes	Yes	8
77	Le Ngoc et al., 2019	Yes	No	Yes	Yes	Yes	Yes	Yes	Yes	Yes	8
78	Li et al., 2014	Yes	No	Yes	Yes	Yes	Yes	Yes	Yes	Yes	8
79	Lioznov et al., 2016	Yes	No	Yes	Yes	Yes	Yes	Yes	Yes	Yes	8
80	Minh 2015	Yes	Unclear	Yes	Yes	Yes	Yes	Yes	Yes	Yes	8
81	Nadol et al., 2016	Yes	Yes	Yes	Yes	Yes	Yes	Yes	Yes	Yes	9
82	Nguyen et al., 2016	Yes	No	Yes	Yes	Yes	Yes	Yes	Yes	Yes	8
83	Nguyen et al., 2018	Yes	Yes	Yes	Yes	Yes	Yes	Yes	Yes	Yes	9
84	Noppornpanth et al., 2006	Yes	No	Yes	Yes	Yes	Yes	Yes	Yes	Yes	8
85	Pham et al., 2009	Yes	No	Yes	Yes	Yes	Yes	Yes	Yes	Yes	8
86	Pham et al., 2011	Yes	No	Yes	Yes	Yes	Yes	Yes	Yes	Yes	8
87	Song et al., 1994	Yes	No	Yes	Yes	Yes	Yes	Yes	Yes	Yes	8
88	Tanimoto et al., 2010	Yes	No	Yes	Yes	Yes	Yes	Yes	Yes	Yes	8
89	Tokita et al., 1994	Yes	No	Yes	Yes	Yes	Yes	Yes	Yes	Yes	8
90	Tran et al., 2003	Yes	No	Yes	Yes	Yes	Yes	Yes	Yes	Yes	8

* **1.** Appropriate sampling frame to address target population, **2.** Appropriate sampling way of study participants, **3.** Adequate sample size, **4.** Detail description of study participants and settings, **5.** Data analysis with sufficient coverage of identified sample, **6.** Use of valid methods to identify the condition, **7.** Standard, reliable way of measurement of condition for all participants, **8.** Availability of appropriate statistical analysis, **9.** Adequate response rate and management of low response rate
