# Supplementary material for: Prevalence of HCV genotypes and subtypes in Southeast Asia: A systematic review and meta-analysis
Source: PLoS One. 2021 May 20;16(5):e0251673. doi: 10.1371/journal.pone.0251673 (PMC8136688; doi:10.1371/journal.pone.0251673)
Supplement: S1 File — (PDF) [file pone.0251673.s019.pdf]

Search strategy

Pubmed

(((“hepatitis c”[Title/Abstract]) OR (HCV[Title/Abstract])) AND ((genotype[Title/Abstract]) OR (genotypes[Title/Abstract]))) AND ((((((((((((Brunei[Title/Abstract]) OR (Cambodia[Title/Abstract])) OR (“East Timor”[Title/Abstract])) OR (Timor-Leste[Title/Abstract])) OR (Indonesia[Title/Abstract])) OR (Laos[Title/Abstract])) OR (Malaysia[Title/Abstract])) OR (Myanmar[Title/Abstract])) OR (Philippines[Title/Abstract])) OR (Singapore[Title/Abstract])) OR (Thailand[Title/Abstract])) OR (Vietnam[Title/Abstract]) OR ("Southeast asia"[Title/Abstract])))

Scopus

TITLE-ABS(“hepatitis c” OR HCV) AND TITLE-ABS(genotype OR genotypes) AND TITLE-ABS(Brunei OR Cambodia OR “East Timor” OR Timor-Leste OR Indonesia OR Laos OR Malaysia OR Myanmar OR Philippines OR Singapore OR Thailand OR Vietnam OR "Southeast asia")

ScienceDirect

(“hepatitis c” OR HCV) (genotype or genotypes) (Brunei OR Cambodia OR “East Timor” OR Timor-Leste OR Indonesia OR Laos OR Malaysia OR Myanmar OR Philippines OR Singapore OR Thailand OR Vietnam OR "Southeast asia")

Google Scholar

allintitle(“hepatitis c” OR HCV) (genotype or genotypes) (Brunei OR Cambodia OR “East Timor” OR Timor-Leste OR Indonesia OR Laos OR Malaysia OR Myanmar OR Philippines OR Singapore OR Thailand OR Vietnam OR "Southeast asia")

Web of Science

(TI=“hepatitis c” OR TI=HCV) AND (TI=genotype OR TI=genotypes) AND (TI=Brunei OR TI=Cambodia OR TI=“East Timor” OR TI=Timor-Leste OR TI=Indonesia OR TI=Laos OR TI=Malaysia OR TI=Myanmar OR TI=Philippines OR TI=Singapore OR TI=Thailand OR TI=Vietnam OR TI="Southeast asia")
